# Mechanistic strategies of microbial communities regulating lignocellulose deconstruction in a UK salt marsh

**DOI:** 10.1186/s40168-020-00964-0

**Published:** 2021-02-17

**Authors:** Daniel R. Leadbeater, Nicola C. Oates, Joseph P. Bennett, Yi Li, Adam A. Dowle, Joe D. Taylor, Juliana Sanchez Alponti, Alexander T. Setchfield, Anna M. Alessi, Thorunn Helgason, Simon J. McQueen-Mason, Neil C. Bruce

**Affiliations:** 1 Centre for Novel Agricultural Products, Department of Biology, University of York, York, YO10 5DD UK; 2 Bioscience Technology Facility, Department of Biology, University of York, York, YO10 5DD UK; 3 Department of Biology, University of York, York, YO10 5DD UK; 4 School of Chemistry and Biosciences, University of Bradford, Bradford, West Yorkshire BD7 1DP UK

**Keywords:** Salt marsh, Lignocellulose, CAZyme, Carbon cycling, Carbohydrate esterase, CE1, Proteomics, Transcriptomics, Community profiling

## Abstract

**Background:**

Salt marshes are major natural repositories of sequestered organic carbon with high burial rates of organic matter, produced by highly productive native flora. Accumulated carbon predominantly exists as lignocellulose which is metabolised by communities of functionally diverse microbes. However, the organisms that orchestrate this process and the enzymatic mechanisms employed that regulate the accumulation, composition and permanence of this carbon stock are not yet known. We applied meta-exo-proteome proteomics and 16S rRNA gene profiling to study lignocellulose decomposition in situ within the surface level sediments of a natural established UK salt marsh.

**Results:**

Our studies revealed a community dominated by *Gammaproteobacteria*, *Bacteroidetes* and *Deltaproteobacteria* that drive lignocellulose degradation in the salt marsh. We identify 42 families of lignocellulolytic bacteria of which the most active secretors of carbohydrate-active enzymes were observed to be *Prolixibacteracea*, *Flavobacteriaceae*, *Cellvibrionaceae*, *Saccharospirillaceae*, *Alteromonadaceae*, *Vibrionaceae* and *Cytophagaceae*. These families secreted lignocellulose-active glycoside hydrolase (GH) family enzymes GH3, GH5, GH6, GH9, GH10, GH11, GH13 and GH43 that were associated with degrading *Spartina* biomass. While fungi were present, we did not detect a lignocellulolytic contribution from fungi which are major contributors to terrestrial lignocellulose deconstruction. Oxidative enzymes such as laccases, peroxidases and lytic polysaccharide monooxygenases that are important for lignocellulose degradation in the terrestrial environment were present but not abundant, while a notable abundance of putative esterases (such as carbohydrate esterase family 1) associated with decoupling lignin from polysaccharides in lignocellulose was observed.

**Conclusions:**

Here, we identify a diverse cohort of previously undefined bacteria that drive lignocellulose degradation in the surface sediments of the salt marsh environment and describe the enzymatic mechanisms they employ to facilitate this process. Our results increase the understanding of the microbial and molecular mechanisms that underpin carbon sequestration from lignocellulose within salt marsh surface sediments in situ and provide insights into the potential enzymatic mechanisms regulating the enrichment of polyphenolics in salt marsh sediments.

Video Abstract

**Supplementary Information:**

The online version contains supplementary material available at 10.1186/s40168-020-00964-0.

## Introduction

Salt marshes are highly productive intertidal ecosystems that generate an abundance of organic carbon in the form of lignocellulose, where net aerial primary productivity often exceeds 1–2 kg C m^−2^ year^−1^ [1–3]. This productivity is intrinsically linked to organic carbon burial rates, estimated to be 57–245 g C m^−2^ year^−1^ [4–6]. This indicates that salt marshes are among the most effective carbon sequestering ecosystems per unit area on the planet with a total estimated sequestration capacity of 4.8 to 87.2 Tg C year^−1^ [7] despite occupying only 22,000–400,000 km^2^ [6–8]. These processes contribute to an increasing pool of inaccessible carbon as the salt marsh accretes. Organic carbon is introduced into the ecosystem as grass lignocellulose which represents the major component of surface to shallow sub-surface level carbon [9]. Furthermore, the composition of organic carbon changes with depth, with an enrichment in persistent lignin derivatives while polysaccharides are lost [10, 11]. Deposited lignin is subject to passive leaching of soluble and often biologically available phenols which diffuse throughout the sedimentary column, adding further recalcitrance to the remaining phenolics, degradation of which is suppressed in anoxic conditions [12–14]. Traditionally, primary productivity, surface area, sediment deposition and transport rates, leaching and sorption govern carbon capture in coastal sediments [15]. Mineral protection and preferential retention of recalcitrant organic carbon are major themes governing carbon sequestration [16]; however, throughout this natural biogeochemical carbon processing, microbial mechanisms of carbon transformation are present and likely operate as a system-level decomposition process that influences the permanence of lignocellulose and stored carbon in marsh sediments. Currently, this process is orchestrated by an undefined consortium of organisms prior to entry into stable deeper sediments where this material persists for millennia [6, 17].

Lignocellulose is a strong fibre composite material which provides mechanical support and the vessels for long distance water transport in plants and is highly resistant to degradation. It is a macromolecular complex formed from cellulose microfibrils embedded in a matrix of branched polysaccharides known as hemicellulose. This polysaccharide complex is interpenetrated and sealed by lignin, a phenolic heteropolymer, making lignocellulose more hydrophobic and difficult to degrade enzymatically [18]. The sheer abundance of lignocellulose in the terrestrial biosphere, along with its complexity and recalcitrance to digestion has led to the evolution of a diverse range of lignocellulolytic enzymes across the tree of life [19]. Yet very little is known about the factors that regulate lignocellulose decomposition in salt marshes despite large annual inputs into these systems as microdetritus that is predominantly retained and degraded on site [9, 20, 21].

The dominant flora in salt marsh ecosystems is perennial such as *Spartina* species. Dieback of these plants introduces vast quantities of lignocellulosic biomass into the marine environment. The first phase of decay occurs during dieback where terrestrial fungal plant pathogens, usually mycelial *Ascomycetes*, attack the senescent plant biomass [22, 23]. These fungi target standing senescent tissue that resides aboveground in a terrestrial setting and act to break open the plant cell walls as a means to access the nutritionally rich cell contents leading to the resultant infected tissues becoming nitrogen depleted and lignocellulose enriched [24–26]. The senescent standing tissue then weakens, and the lignocellulose enriched biomass detaches from the root-rhizome becoming deposited onto the sediment surface where it transitions into a predominantly marine environment with significantly greater and more variable physico-chemical pressures than terrestrial zones [27]. Here, it redistributes around the salt marsh surface or aggregates on the strandline where it is subject to a different phase of decay.

Studies have established degradation rates of deposited lignocellulose in situ at surface levels using litterbag methodologies [9, 20, 28–31]; however, very little is known about the microbial framework that regulates this decomposition or the enzymatic mechanisms employed to deconstruct the complex lignocellulosic substrate. In vitro studies have suggested that bacteria, such as *Cyclobacteriaceae*, *Desulfobacteraceae*, *Flavobacteriaceae*, *Halomonadaceae*, *Oceanospirillales*, *Pseudomonadaceae* and *Spirochaetaceae*, are involved in lignocellulose degradation in this environment with fungi becoming competitively displaced [32–34]. Beyond this, our understanding of the functional groups involved in the decomposition process and the biocatalytic strategies they employ to achieve this are poorly understood. In vitro studies are divorced from environmental factors and the findings require cautious interpretation, as results cannot be directly extrapolated into the context of ecosystem processes. Additionally, the function of an organism cannot be determined by its presence or the presence of a gene as this only deduces a potential propensity to function.

**Fig. 1:**
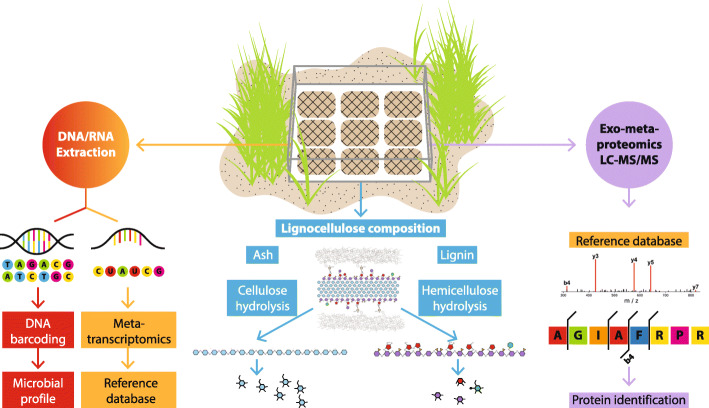
Schematic representation of the integrated omics approach undertaken in this study

Direct monitoring of ecological processes in situ has the potential to capture functional, molecular and phylogenetic information at their environmental interface. To identify the microbial community that regulates the initial decomposition of introduced lignocellulose at the surface level, we applied meta-exo-proteome proteomics, ribosomal 16S rRNA gene phylogenetic profiling and lignocellulose composition analysis to *Spartina anglica* biomass in litterbags in situ along a 300-m transect within an established salt marsh (Welwick, UK) for 16 weeks (Fig. 1, Additional file 1: Figure S1). We identify lignocellulolytic enzymes from the meta-exo-proteome, ascertain the taxonomic origin to identify functional groups and determine the mechanistic strategies they employ to depolymerize lignocellulose.

## Materials and methods

### Experimental design

The field experiment was conducted in Welwick salt marsh, Hull, Humber estuary, UK, 53° 38′ 55″ N, 0° 01′ 19″ E from 16 July 15 to 6 November 15 (Additional file 1: Figure S1). To mimic natural lignocellulose cycling, senescent aboveground *Spartina anglica* biomass was collected prior to deposition during winter dieback in February–March 2015 on an adjacent intertidal mud flat (Cherry Cobb sands, Humber estuary, Hull, UK). The biomass was washed free of sediment, dried at 65 °C for 48 h and size fractionated with a Retsch Cutting Mill SM 300 at 2300 rpm. The final biomass fraction consisted of 80% of > 1.12 mm fraction and 20% < 1.12 mm to > 500 μm fraction. Nylon 66 monofilament woven bags (“litterbag”) (18.5 cm × 18.5 cm) of aperture size 200 μm were filled with 50 g of biomass and sealed with 100% polyester thread.

Bags were placed in a 3 × 3 conformation in five stainless steel cages (711.2 mm × 711.2 mm × 63.5 mm) with 25-cm legs that were interspersed by 75 m along a 300-m parallel elevation transect, defined by plant zonation patterns (dominance of *Spartina anglica*, *Puccinellia maritima* and *Salicornia europaea*). Prior to deployment, the under canopy was removed and the cages placed with the bags interfacing with the sediment to facilitate crosstalk to mimic surface-interfacing detritus and mapped to position with GPS coordinates (Additional file 1: Table S1).

Sampling was performed by removing a single litterbag from each cage. During deployment, the uppermost 1–5 mm of sediment surrounding the cages were sampled to act as a non-lignocellulose enriched sediment day 0 outgroup control. Sampling was randomised a priori and occurred weekly for the first 6 weeks and thereafter at week eight, ten and 16 for a total of 46 samples (including the day 0 outgroup). Sampling began at the point of low tide and was completed within 2 h. Sampled bags were kept at 4 °C during transport and processing began within 4 h of harvest.

### DNA and RNA extraction

Each biological replicate at each time point (week one–six, eight, ten and 16) were treated independently. Harvested biomass was equilibrated twice with 40 mL ice cold 0.5x PBS pH 8.15 and centrifuged for 10 min at 4500×*g*. Five 0.5 g biomass aliquots per litterbag (per cage; 25 total per week) were taken forward for DNA and RNA extraction. The five 0.5 g biomass aliquots were added to screw cap tubes (2 mL) containing 0.5 g 0.5 mM glass beads (Sigma G9268) and 0.5 g 0.1 mM glass beads (Sigma G8893). Cetyl trimethylammonium bromide buffer (0.5 mL) containing 10% CTAB (m/v) in 0.7 M NaCl, 240 mM potassium phosphate pH 8 and 0.1% β-mercaptoethanol and 0.4 mL phenol/chloroform/isoamyl alcohol (25:24:1) pH 8 were added. The samples were homogenised in a TissueLyser II (Qiagen) for 2 × 2.5 min at 30/s. The tubes were then centrifuged at 4 °C at 16,500×*g* for 15 min. The aqueous phase was transferred to a new tube and an equal volume of chloroform to isoamyl alcohol (24:1) was added and centrifuged as previously and three 7.5 g aliquots of decaying *Spartina* biomass per litterbag (one litterbag per each of the five cages for a total of 15 aliquots per week; only for weeks one, three, five and ten) were taken forward for protein extraction following Alessi et al. [35]. The aqueous phase was precipitated for 16 h at 4 °C with two volumes of PEG precipitation solution containing 20% (w/v) PEG8000 (Sigma) in 1.6 M NaCl. The nucleic acid pellet was collected by centrifugation as above for 30 min at 4 °C. The pellet was washed twice in 75% ethanol. Total RNA from weeks one, three, five and ten (the same samples used to extract the proteins within the meta-exo-proteome) were taken forward for metatranscriptomic processing.

### Meta-exo-proteome extraction

Each biological replicate in the protein extraction was treated independently. Per week, for each of the five cages, three aliquots (only for weeks one, three, five and ten, the same weeks utilised for metatranscriptome extraction to generate complimentary paired-in-time databases) were taken forward for protein extraction to generate meta-exo-proteome libraries. Each 7.5 g aliquot of harvested biomass was washed twice with 40 mL ice cold 0.5x PBS pH 8.15 and centrifuged for 10 min at 4500×*g*. The extracellular and transmembrane proteins were labelled in triplicate and 2.5 g biomass aliquots for each of the biological replicates were resuspended in 10 mM EZ-link-Sulfo-NHS-SS-biotin (Thermo Scientific #21331) in 0.5x PBS and incubated at 4 °C for 1 h. The biomass was centrifuged for 10 min at 4500×*g* as above, the supernatant was discarded and the biotinylation reaction was quenched with 25 mL 50 mM Tris-HCl pH 8 and incubated for 30 min at 4 °C. Excess Tris-HCl and residual biotin was removed with two washes with 20 mL ice cold 0.5X PBS pH 8 with centrifugation steps for 5 min at 4500×*g*.

Proteins were extracted from the biomass with 10 mL 2% (w/v) SDS pre-heated to 60 °C and incubated for 1 h. The supernatant was extracted, and the proteins were precipitated with five volumes of pre-chilled (− 20 °C) 100% acetone and incubated at − 20 °C for 16 h. Precipitated proteins were pelleted by centrifuging at 4500 rpm for 20 min and the residual acetone was discarded. The pellets were air dried and resuspended in 1 mL 0.1% SDS in 1x PBS, filtered through 0.22 μm syringe driven filter units and loaded onto 1 mL HiTrap Streptavidin HP columns (GE Healthcare #17-5112-01) and incubated for 1 h at 4 °C. The proteins were eluted with 1 mL 50 mM dithiothreitol (DTT) in 1x PBS, the column was incubated for a further 1 h and eluted again, this was performed three times and the first two 1 mL fractions were pooled.

The protein fractions were desalted and buffer exchanged into H_2_O using 5 mL Zeba™ Spin 7 k MWCO columns (Thermo 89882) according to the manufacturer’s protocol. To concentrate, the buffer exchanged protein was frozen in liquid nitrogen, lyophilised using a Heto PowerDry LL3000 Freeze Dryer (Thermo) and resuspended in 210 μL H_2_O. All five biological replicates for each time point were pooled in equal concentrations. The proteins were stored for LC-MS/MS analysis by solubilising in NuPAGE LDS sample buffer (Life Technologies) and incubating at 70 °C for 10 mins prior to a short (6 min) run into a 7-cm NuPAGE Novex 10% Bis-Tris Gel (Life Technologies) at 200 V. The gels were stained with SafeBLUE protein stain (NBS biologicals) for 1 h before de-staining with H_2_O for 1 h. The stained gels were sliced into 1-mm^2^ fragments and stored at − 20 °C prior to LC-MS/MS analysis.

### Meta-exo-proteomics, protein identification, functional annotation and taxonomic origin

To generate paired-in-time reference metatranscriptome databases, total extracted nucleic acids from each biological replicate were pooled in equal ratios for each time point (week one, three, five and ten) and DNA depleted. Messenger RNA (mRNA) was enriched by depleting ribosomal RNA (rRNA) using Ribo-Zero™ Magnetic Epidemiology rRNA removal kit (RZE1224/MRZ11124C; Illumina). RNA-seq libraries were prepared using a NEBnext RNA Ultra Library preparation kit with NEBnext single 6 bp indexing primers (New England BioLabs, Herts, UK) and pooled in equimolar ratios. The pooled RNA-seq library was spiked with 1% PhiX and sequenced on a single lane of an Illumina HiSeq 3000 2 × 150 base pair chip. Sequencing resulted in 82 966 97, 99 319 32, 95 318 91 and 105 517 252 raw reads for the metatranscriptomics databases for week one, three, five and ten respectively (383,122,461 reads in total); statistics for the four individual metatranscriptomic databases and totals are available in Additional file 1: Table S2.

To leverage the depth of sequencing and capitalise on the diversity within the temporally interspersed metatranscriptomes maximise protein identification, the metatranscriptomic databases for week one, three, five and ten were concatenated into a single master metatranscriptome. Raw reads were searched against Silva_115 database to identify ribosomal RNA (rRNA) genes using the Bowtie2 software package [35, 36]. Orphan reads in the paired reads, rRNA reads and poor-quality sequences were removed with the ngsShoRT software [37]. Dereplicated libraries were assembled de novo with the Trinity software package [38]. Read counts and gene abundance were obtained with the Trinity utility programs. The de novo assembled metatranscriptomic databases contained 29,938,868 contiguous sequences (contigs). Contigs ≤ 500 bp were filtered, split into open reading frames (ORFs) using Emboss GETORF (http://www.bioinformatics.nl/cgi-bin/emboss/getorf) that were ≥ 300 bp and includes alternative initiation codons and dereplicated resulting in 2,400,360 unique ORFs within the master metatranscriptome.

To generate paired-in-time exo-meta-proteome databases, biological replicates at week one, three, five and ten were pooled and protein identification was performed in triplicate for each pool at each time point (*N* = 3 for each of week one, three, five and ten). Tryptic digestion was performed for in-gel proteins post reduction with DTE and *S*-carbamidomethylation with iodoacetamide. Gel pieces were washed twice with 50% (v:v) aqueous acetonitrile containing 25 mM ammonium bicarbonate and finally washed with acetonitrile and then dried. Modified porcine trypsin (Promega, Southampton, UK) was dissolved in 50 mM acetic acid and diluted with 25 mM ammonium bicarbonate to 0.02 μg/μL. 25 μL of trypsin solution was added and incubated for 10 min before adding 25 mM ammonium bicarbonate to submerge to gel pieces and incubated further for 16 h at 37 °C. Three washes were performed with 50% (v:v) aqueous acetonitrile containing 0.1% TFA (v:v), dried and reconstituted in aqueous 0.1% trifluoroacetic acid (v:v).

The acquisition of peptide spectra was performed in triplicate for each time point and was achieved using a nanoLC system interfaced with a maXis HD LC-MS/MS system and a CaptiveSpray ionisation source (Bruker Daltonics, Coventry, UK). Positive ESI-MS and MS/MS spectra were acquired using AutoMSMS mode. Instrument control, data acquisition and processing were performed using Compass 1.7 software (microTOF control, Hystar and DataAnalysis, Bruker Daltonics). Instrument settings were the following: dry gas, 3 L/min; ion acquisition range, *m/z* 150–2000; MS/MS spectra rate, 5 Hz at 2500 cts to 20 Hz at 250,000 cts; quadrupole low mass, 300 *m/z*; cycle time, 1 s; ion spray voltage, 1450 V; collision RF, 1400 Vpp; transfer time, 120 ms; MS spectra rate, 5 Hz; dry gas temperature, 150 °C; absolute threshold 200 counts; preferred charge states 2–4; and singly charged ions excluded. A single MS/MS spectrum was acquired for each precursor and former target ions were excluded for 0.8 min unless the precursor intensity increased fourfold.

Our approach of shotgun LC-MS/MS-based proteomics allows in-depth proteomic analysis but is only effective if the peptide spectra can be matched to a corresponding sequence database. Because the salt marsh environment has been little explored at the molecular level, we used the metatranscriptome libraries, that were generated at the same time points (paired-databases in time) as the proteomic studies, as reference libraries to map peptide spectra to their originating sequence. We concatenated the four metatranscriptomic databases (week one, three, five and ten) into a master database to capitalise on the diversity within the temporally interspersed metatranscriptomes and used this gene expression data to identify meta-exo-proteome proteins from peptide spectra, shedding new light on the communities of microbes in this environment and their activities.

To identify proteins from LC-MS/MS spectra, peptide spectra generated from the digested meta-exo-proteome proteins were mapped back to originating sequences in the ORF library generated from the concatenated metatranscriptomic assemblies. Firstly, redundant sequences in the ORF database were removed by leveraging non-redundant sequences in an initial round of high stringency searching (*p* = 0.05) against 21 subsets of ~ 115 000 sequences (252 searches total), followed by the concatenation of sequence hits into a secondary “true hit” database (containing 42,894 sequences) with minimal redundancy, the final search against the true hit database (*p* = 0.1) yielded 11,268 unique proteins; individual peptide spectral matches were filtered to require expect scores of 0.1 or better. Peptide spectra generated with LC-MS/MS were cross-referenced with ORF sequences using Mascot version 2.5.1 (Matrix Science Ltd.), through the ProteinScape interface version 2.1 [39]. The search criteria for both searches were + 2/+ 3/+ 4 peptide charge, peptide tolerance ± 10 ppm, modifications, carbamidomethyl and oxidation. Analysis was performed using the quantitative exponentially modified protein abundance index (emPAI) [40]. emPAI values for each protein were then normalised to generate the molar percentage.

dbCAN was used to identify carbohydrate-active enzymes (CAZyme) within the meta-exo-proteome and the metatranscriptomic databases using HHMER3 [41]. The meta-exo-proteome was also searched against the NCBI non-redundant protein database (NR_prot; 1:62) using BLAST+ (BlastP) version 2.2.31 with an expect value threshold of 1e^−5^ [42]. The resulting best-hit was obtained for each protein in the meta-exo-proteome and NCBI Accession and TaxID database was compiled and the most likely taxonomic origin of these proteins were established using tools within the Environment for Tree Exploration (ETE) version 3 toolkit [43]. To delineate functional members of the microbial community associated with the *Spartina* biomass, we cross-referenced the 16S rRNA gene phylogenetic profile with the taxonomic origin of the meta-exo-proteome proteins.

### 16S rRNA gene and ITS2 amplicon sequencing and analyses

Biological replicates were treated independently (*N* = 5) for each of the 9 time points (week one–six, eight, ten, 16 and the day 0 outgroup for a total of 46 data points). Total extracted nucleic acids were RNAse A treated in triplicate. The ribosomal 16S rRNA gene V4 region was targeted with primers, 515f-Y GTGYCAGCMGCCGCGGTAA (5′–3′) [44] and 806R GGACTACNVGGGTWTCTAAT (5′–3′) [45]. The internal transcribed region 2 (ITS2) region was targeted with primers, fITS7 GTGARTCATCGAATCTTTG (5′–3′) [46] and ITS4ngs TCCTSCGCTTATTGATATGC (5′–3′) [47]. Cluster identification was enhanced with a random dodecamer sequence NNNHNNNWNNN (5′–3′) prepended to the forward primer [48].

16S rRNA gene polymerase chain reactions (PCR) were performed in 25 μL volumes containing 200 μM dNTPs, 0.5 μM 515fY-MN, 0.5 μM 806rMN, 50 ng gDNA, 0.5 U Phusion HF polymerase (#M0530) and 1x Phusion HF Buffer. Thermocycling conditions included an initial denaturation at 98 °C for 30 s, followed by 28 cycles of 98 °C for 10 s, 53 °C for 30 s and 72 °C for 15 s and 72 °C for 10 min. ITS2 PCR were performed as above with thermocycling conditions including an initial denaturation at 98 °C for 30 s, followed by 34 cycles of 98 °C for 10 s, 57 °C for 30 s and 72 °C for 20 s, with final extension at 72 °C for 5 min. Indexing was performed using the Nextera XT™ library preparation kit (Illumina FC-131-1001). The libraries were pooled in equimolar concentrations to 4 nM, spiked to 1% PhiX and run on a MiSeq 250 bp × 2 cartridge (MiSeq Reagent Kit v2 (500 cycles) MS-102-2003, Illumina).

The generated 16S rRNA genes libraries averaged 54,929 sequences. Fastq merging was performed with Vsearch version 1.11.1 [49]. The generated ITS2 libraries averaged 50,843 sequences and were processed with ITSx [50] to filter non-fungi sequences. The resulting fungi only ITS2 libraries averaged 28,972 sequences. The primer sequences were trimmed using Cutadapt (version 1.11.). Sequences were trimmed to global lengths of 250 bp using Usearch (version 9, -fastx_truncate) [51]. Amplicon profiles were dereplicated, purged of singletons, assigned abundance and sorted by size using Usearch (version 7, -derep_fulllength) [51]. Clustering was performed using the UPARSE algorithm [52], with concurrent de novo chimaera detection using Usearch (version 9, -cluster_otus) with a 97% identity threshold resulting in 5122 non-chimeric operational taxonomic units (OTUs) that were taken forward for analysis. Representative sequences for each OTU were then mapped to original sequences using Usearch (version 7, -usearch_global). Taxonomy was assigned with QIIME [53] (version 1.9, assign_taxonomy.py) using SILVA 132 [35] for the 16S rRNA libraries and UNITE v7.1 [54]. Rarefaction analysis [53] displayed curves that begin to reach asymptotic levels, indicating sufficient depth for analysis but not complete diversity coverage (Additional file 1: Figure S2). The taxonomy of any unassigned OTUs (*N* = 610), using UNITE in the ITS2 libraries were further classified using BLASTn against the GenBank non-redundant nucleotide database. Non-fungal OTUs were discarded and missing taxonomies of on target OTU sequences were manually curated (*N* = 393) resulting in a total of 920 fungal OTUs which were subsequently analysed. Fungal OTUs were classified into functional guilds using FUNGuild [55] which assigned a functional guild to 419 OTUs from 724 matches of the original 920, this represented 51.4 ± 2.12% mean OTU abundance across all time points and was taken forward for analysis. All commands for the analysis pipeline are available in Additional file 1: Table S3.

### Determining highly productive groups

A productivity index was used to elucidate taxonomic groups with disproportionately greater CAZyme production per unit abundance, given by log_10_(∑ $\overline{\mathrm{x}}$ mol%/abundance). Disproportionately productive groups were determined as those with an index > 0.3 in at least 1 observation.

### Network associations

Network associations between meta-exo-proteome CAZyme classes and taxonomic classes were constructed by grouping annotated domains (≤ 1e^−10^) into CAZyme classes by average ∑ $\overline{\mathrm{x}}$ mol% across the entire time course and connecting these nodes to the taxonomic classes the domains originated from. Classes are presented by their mean ∑ $\overline{\mathrm{x}}$ mol% output. Taxa < 0.025 ∑ $\overline{\mathrm{x}}$ mol% with ≤ 5 edges (connections) and CAZyme classes < 1.25 × 10^−3^ ∑ $\overline{\mathrm{x}}$ mol% were filtered for clarity. Plots were generated with NetworkX [56].

### Biomass composition analysis

Biomass was washed free of sediment through 100 μm mesh with free flowing dH_2_O. Total biomass was measured as the mass balance of lyophilised material. Ash was determined with 1 g of biomass incubated 600 °C for 24 h. Matrix polysaccharides were measured using triflouracetic acid methodology [57]. Cellulose was subsequently determined using the Updegraff and Saeman hydrolysis [58, 59]. Lignin was measured as acetyl bromide soluble lignin [60] using a previously cited extinction coefficient of 17.75 for grasses [61].

### Statistics

One-way ANOVAs and Tukey’s HSD tests were performed using SciPy [62] and Scikit [63], respectively. All data were assessed for normality using the Shapiro-Wilk test. Statistical analyses were performed on non-normalised data.

## Results

### Functional assignment of the meta-exo-proteome

To identify the lignocellulolytic enzymes involved in biomass breakdown, we employed a metaproteomic analysis of extracellular proteins (meta-exoproteome), accomplished by an affinity tagging process using a membrane-impermeable biotinylation tag [64]. Because lignocellulose is an insoluble macromolecule, it generally has to be broken down by extracellular enzymes. Many of the enzymes involved adhere to the lignocellulose or the microbe, and the use of surfactants to extract them leads to cell lysis and contamination with intracellular proteins. The tagging approach avoids the problem of intracellular contaminants, allowing a focus on extracellular and cell surface proteins.

Annotation of the transcriptome revealed 103 CAZyme families (≤ 1e^−5^ and transcripts per million (TPM) ≥ 1) across 44,334 ORFs (excluding glycosyl transferases), the total proportion of CAZYmes across all transcriptomic databases was 429.27 ± 62.16 TPM (Additional file 1: Figure S3). Proteomic analysis identified 11,268 proteins within the meta-exo-proteome, of which 320 (≤ 1e^−10^) were annotated as putative carbohydrate-active domains (CAZyme) within 252 peptide matching ORFs across 81 CAZyme families. Families present within the metatranscriptomic databases that were absent from the meta-exo-proteomes were largely families not specific to lignocellulose degradation or families usually associated with core intracellular activities (AA6, CE14, GH32, GH57, GH73, GH92 and GH108) or CAZyme families containing enzymes with both intracellular and extracellular localisations (GH1, GH2, CE7) with the exception of a small subset of predominantly pectin-targeting CAZymes: CE2, CE7, CE8, CE12, CE15, GH28 and GH105 and AA4 (Additional file 1: Figure S3). Instead, the exo-meta-proteome predominantly consisted of pectin-targeting CAZYme families CE3, CE4, CE6, PL1, PL4, GH35 and GH43.

**Fig. 2:**
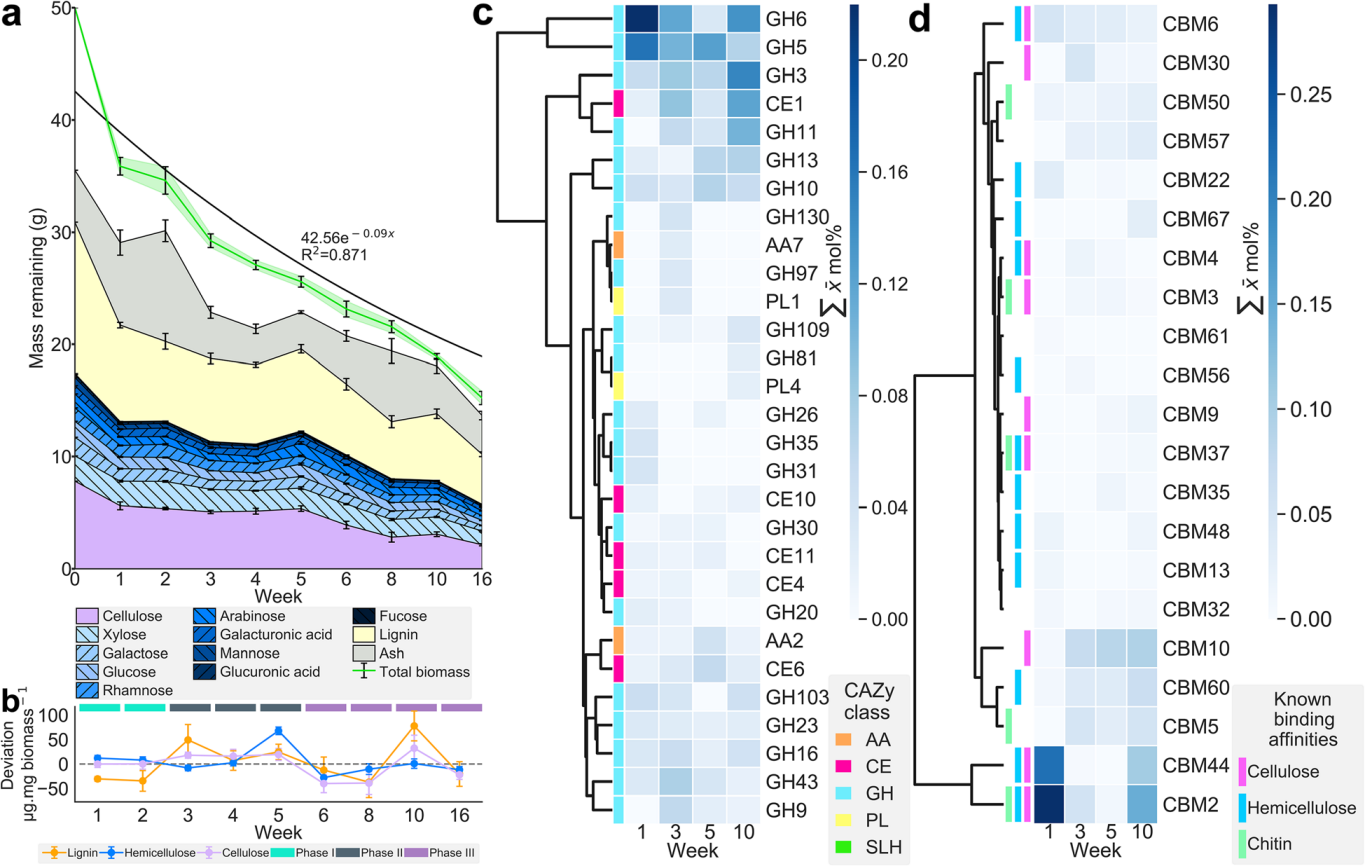
Temporal changes in lignocellulose composition and the distribution of carbohydrate-active enzyme domains within the meta-exo-proteome. **a** Lignocellulose composition of remaining in situ *Spartina anglica* biomass. **b** Rate of compositional change within the lignocellulose displayed as μg mg biomass^−1^, the dashed line represents 0 change; L, lignin; H, hemicellulose; C, cellulose. **c** Euclidean clustering of the enzyme class profile (≤ 1e^−10^), the 30 most abundant classes are displayed; GH, glycoside hydrolase; CE, carbohydrate esterase; AA, auxiliary activity; PL, polysaccharide lyase. **d** Euclidean clustering of the carbohydrate binding domain (CBM) profile (≤ 1e^−10^). Error bars (**a**) represent SE (*n* = 25). Figure plotted with [66]

CAZyme homologues (≤ 1e^−10^) represented only 0.72–0.99 mol% of the total meta-exo-proteome concordant with previous in vitro reports [65]. The meta-exo-proteome CAZyme profile revealed three dominant Euclidean clusters of temporally abundant classes which contain a diverse collection of activities (Fig. 2c). Glycoside hydrolases (GH) were the most abundant class with 37 families identified. GH3, GH5 and GH6 family enzymes were abundant; these classes are typically related to cellulose degradation, many of which were associated to carbohydrate-binding domains (CBMs). The CBM profile of our data highlighted two abundant Euclidean clusters (Fig. 2d); the dominant of which contained CBM2 and CBM44 motifs associated with cellulose and matrix polysaccharide binding and a secondary cluster containing CBM10, CBM5 and CBM60 which have been associated with cellulose, hemicellulose and chitin binding, respectively. Families associated with hemicellulose degradation were abundant, notably GH10, GH11 and GH16 typically associated with xylan degradation.

A rapid loss of dry mass was observed with a reduction of 69% during the 16-week period. The distribution of CAZyme family proteins coupled with the biomass composition revealed successional targeting of the major lignocellulose biopolymers (Fig. 2a, b), that temporally synchronised with the abundance of CAZyme proteins within the meta-exo-proteome. The largest loss in cellulose occurs during the first week, most likely conducted by the highly abundant GH6 and GH5 family enzymes coordinated with CBM2 and CBM44 domains targeting exposed cellulose microfibrils generated as a result of the mechanical fractionation of the *Spartina anglica* biomass, while lignin degradation appears rate limiting (weeks one and two). During weeks three to five, there was an increased rate of matrix polysaccharide loss which corresponds to an increased abundance of GH11, GH10, GH13 and GH43 family enzymes coupled with a concomitant decline in the rate of cellulose hydrolysis, suggesting matrix polysaccharides limited cellulose access. During weeks 6 to 16, the rate of cellulose deconstruction increases and a degradative equilibrium was established.

An interesting finding was that carbohydrate esterases (CE) were more abundant than many GH family enzymes (Fig. 2b), particularly those from family 1 (CE1) that predominantly presented as feruloyl esterases and acetyl xylan esterases. Auxiliary activities (AA) established largely as encompassing oxidative enzymes, while present within the enzymatic profile, were not abundant. We only identified two AA families; AA7 (glucooligosaccharide oxidases and chitooligosaccharide oxidases) which were transiently present during week three and AA2 (containing class II lignin-modifying peroxidases) that were present at low abundances throughout the study.

### Taxonomic affiliation of meta-exo-proteome proteins

Fungi and archaea were poorly represented in our metaproteome annotations and were only responsible for 0.28–1.46 and 0.04–0.2 mol% of the total meta-exo-proteome, respectively. Bacteria produced 99–100 mol% CAZymes. Indeed, within the CAZyme profile, the only notable proteins not of bacteria*/*archaea origin showed homology to Annelida (AA2) and Chlorophyta (AA3) enzymes. This was concordant with the total meta-exo-proteome, of which 66.5–79.5 mol% originated from bacteria*/*archaea.

**Fig. 3:**
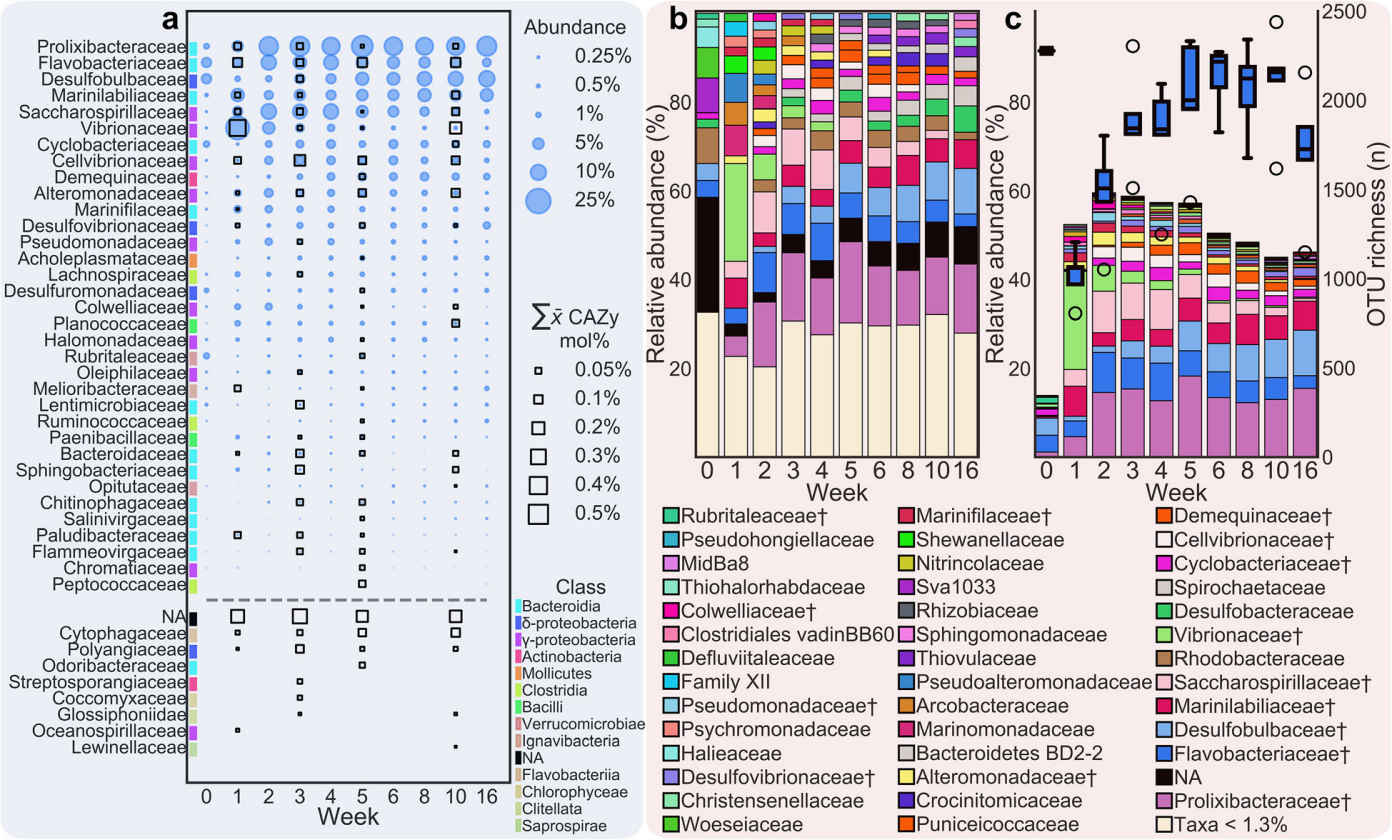
CAZyme-producing taxa at family level resolution and their respective CAZyme contributions. Microbiome and proteomic data is displayed as the mean of *n* = 5 and *n* = 3 respectively. **a** Distribution of CAZyme-producing lineages with respective CAZyme productivity (≤ 1e^−10^), taxa below the dashed line were not identified in the community profile. **b** Bacteria profiles elucidated from 16S rRNA gene sequence homology, each time point is the mean of five biological replicates. **c** CAZyme productive bacteria profile, the non-CAZyme productive taxa have been filtered, boxes display OTU richness, no further filtering was undertaken for these data. NA, not assigned; dagger indicates CAZyme producer. Figure plotted with [66]

Proteins that originated from families also identified in the 16S rRNA gene-derived community profile accounted for 75 ± 6.9% CAZyme mol%. The results indicate *Proteobacteria* and *Bacteroidetes* are the dominant producers of lignocellulolytic enzymes (Fig. 3a). *Gammaproteobacteria* and *Deltaproteobacteria* were responsible for 39.03 ± 13.65% and 7.48 ± 3.95% of total CAZyme mol%, respectively, while *Bacteroidia*, *Flavobacteriia* and *Cytophagia* were responsible for 12.45 ± 6.30%, 9.25 ± 2.55% and 7.45 ± 3.03% of the total CAZyme mol%, respectively. This is concordant with the 16S rRNA gene abundance of these two phyla, which is maintained at 78.43 ± 4.10%. Investigations revealed *Alteromonadaceae* (*Alteromonas*, *Rheinheimera and Catenovulum*), *Vibrionaceae* (*Vibrio*), *Flavobacteriaceae* (predominantly *Lutibacter*, *Wenyingzhuangia* and *Flavobacterium*), *Cellvibrionaceae*, *Saccharospirillaceae* and *Reinekea*, *Prolixibacteraceae* (predominantly *Draconibacterium*, *Prolixibacter* and *Sunxiuqinia*), *Marinilabiliaceae* (*Saccharicrinis*), *Saccharospirillaceae* (*Reinekea*) and *Bacteroidaceae* (*Bacteroides*) as dominant CAZyme producers (Fig. 3). Groups with disproportionately high CAZyme productivity relative to their abundance were revealed as *Bacteroidaceae* (*Bacteroides*), *Paludibacteraceae* (*Paludibacter*), *Flammeovirgaceae* (*Flexithrix*), *Sphingobacteriaceae*, *Melioribacteraceae* (*Melioribacter*), *Chromatiaceae*, *Peptococcaceae* and *Salinivirgaceae* (*Salinivirga*) (Additional file 1: Figure S5). CAZyme productive but poorly resolved genera included *Teredinibacter*, *Sporocytophaga*, *Aquimarina*, *Hyunsoonleella*, *Planococcus*, *Pseudosphingobacterium*, *Desulfosporosinus*, *Formosa*, *Simiduia*, *Sorangium*, *Lentimicrobium*, *Arcticbacter*, *Desulfobulbus*, *Saccharophagus* and *Chitinophaga* (Additional file 1: Figure S4).

Fungi were identified within the sediment and lignocellulosic material but no CAZymes originating from fungi were detected. Significant changes in fungal OTU richness was observed (ANOVA, *F*_8,36_ = 14.95, *p* < 2.29 × 10^−9^) with a significant increase (ANOVA, *F*_1,8_ = 29.17, *p* < 0.0006) between week one and the observed peak during week two from 253 ± 35 to 360 ± 18.54, respectively, before entering a gradual but continuous decline to week 16 (*N* = 180 ± 24.2) (Additional file 1: Figure S11). Fungal taxonomy was poorly resolved with 558 of 920 OTUs identified to class level. Identified fungi were predominately *Ascomycetes* (56.38 ± 4.07%) with a small contribution from *Basidiomycota* (5.22 ± 1.57%); however, in the day 0 sediment *Rozellomycota*, *Chytridiomycota* and *Zygomycota* were observed as very minor components.

*Saccharomycetales* and *Pleosporales* were consistently abundant within the lignocellulose associated fungal community throughout the 16 week time course (25.6 ± 3.23% and 10.85 ± 1.74%, respectively) (Additional file 1: Figure S11). Notable components of the early fungal profile included *Hypocreales*, *Capnodiales* and *Tremellales* (weeks one through to three) before rapidly declining and seemingly displaced by *Microascales* which enrich and dominate the profile between week five (1.82 ± 1.2%) and six onwards (10.2 ± 4.45%). Functional classification of these OTUs in terms of nutrient acquisition strategy revealed the dominant guild to be saprotroph, followed by pathotroph-saprotroph (Additional file 1: Figure S12), of which the most prevalent trophic modes were undefined saprotroph which enriched gradually from 32.3% in the day 0 sediment outgroup to 86.1 ± 3.32% at week 16, endophyte-lichen parasite-plant pathogen-undefined saprotroph which were consistent between week two and 16 (20.1 ± 1.91%) and animal pathogen-endophyte-lichen parasite-plant-pathogen-soil saprotroph-wood saprotroph which was a large component only during weeks one to three (13.8 ± 1.72%; Additional file 1: Figure S11), both of which are poorly resolved definitions. Interestingly, modes associated with the turnover of lignocellulosic substrates such as wood saprotroph and leaf saprotroph were more abundant in the day 0 sediment outgroup than in the lignocellulose associated community.

Filtering out non-CAZyme productive lineages revealed a rapid enrichment for CAZyme-producing families relative to the day 0 sediment outgroup. This suggests that within the sediment, a maximum of 13.9% of the bacteria/archaea microbiome at family level functioned as lignocellulose degraders while OTU richness was highest (2277). During the first week within the biomass, we observed an enrichment in CAZyme productive lineages of 3.77 ± 0.11 fold to 52.40 ± 1.51% of the total community while OTU richness declined (1020 ± 65), increasing to 59.56 ± 1.66% in week two (Fig. 3b, c). We observed significant variation in OTU richness over time (ANOVA, *F*_8,36_ = 17.59, *p* < 0.000005), increasing significantly from week one to three and all time points thereafter (Tukey HSD, *p* < 0.015). OTU richness continued to increase toward day 0 outgroup levels while no significant decline in the abundance of CAZyme-producing members was observed during the time course (ANOVA, *F*_8,36_ = 1.78, *p* > 0.114) suggesting the colonisation of diverse heterotrophs and secondary metabolizers. Concordantly, the total CAZyme mol% was not significantly different throughout the time series (ANOVA, *F*_3,8_ = 1.06, *p* > 0.42).

**Fig. 4:**
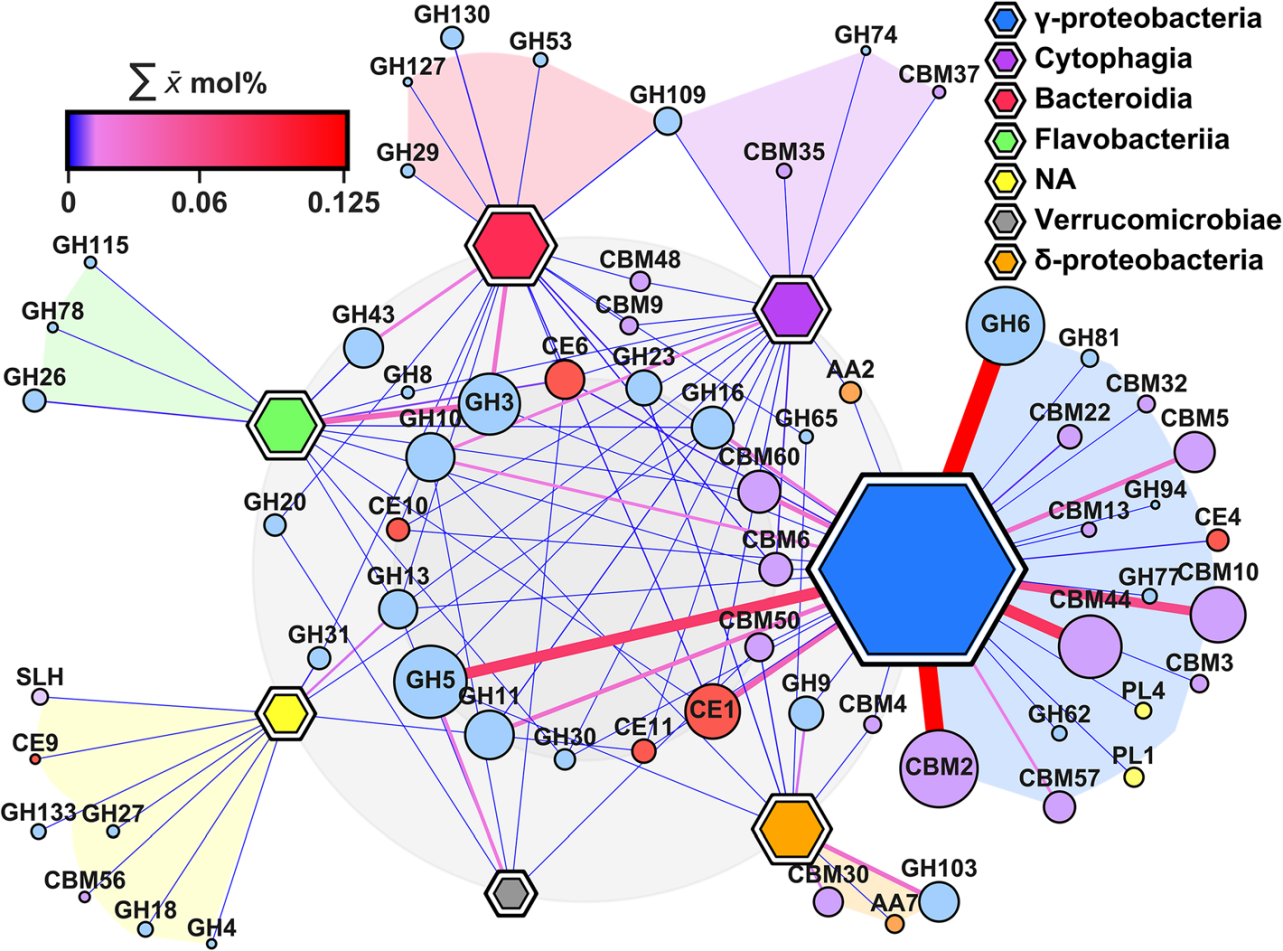
Network associations among meta-exo-proteome CAZyme classes and taxonomic lineages. Data displayed is the mean of the four time points. Node area is proportional to productivity and abundance for taxa and CAZyme class respectively. Edge colour and width is relative to output size. Taxa < 0.025 ∑ $\overline{\mathrm{x}}$ mol% with ≤ 5 edges and CAZyme classes < 1.25 × 10^−3^ ∑ $\overline{\mathrm{x}}$ mol% have been filtered. Glycoside hydrolases (GH) families; blue nodes, carbohydrate esterases (CE) families; red nodes, auxiliary activities (AA) families; orange nodes, polysaccharide lyases (PL) families; yellow nodes, carbohydrate-binding domains (CBM) families; purple nodes. *NA*, not assigned

We noted a degree of congruence between the enzymatic profiles of the most productive groups within *Proteobacteria* and *Bacteroidetes* (Fig. 4, Additional file 1: Figures S4–7). The most abundant CAZyme classes (Fig. 2c, b) with the exception of GH6, CBM2, CBM10 and CBM44 that were produced exclusively by *Gammaproteobacteria*, represented a core suite of activities (Fig. 4) and were produced by multiple divergent lineages, suggesting a common mechanistic strategy was employed by the major CAZyme-producing consortia (Fig. 4).

*Gammaproteobacteria* maintain unparalleled levels of CAZyme production across the time course despite a reduction in their overall abundance. This is due to an enrichment in clades exhibiting high CAZyme production, e.g. *Alteromonadaceae*, *Saccharospirillaceae* and *Vibrionaceae*. *Cellvibrionaceae*, *Alteromonadaceae* and *Saccharospirillaceae* are not abundant in sediments but progressively became major components of the *Gammaproteobacteria* profile in both the community profile and their CAZyme output. *Vibrionaceae* appear transient with peak abundance during week one (22.98 ± 3.27%) which precedes a steady decline (5.25 ± 1.04% and 2.70% ± 0.70% in weeks two and three, respectively), indicating that this clade represent rapid colonisers and opportunistic oligotrophs. *Vibrionaceae* was predominantly comprised of two genera, *Vibrio* and *Photobacterium*. Subsequently, *Vibrionaceae* appears to be outcompeted by *Alteromonadaceae*, *Saccharospirillaceae* (*Reinekea*) and *Cellvibrionaceae* (predominantly *Marinagarivorans*), accounting for much of the decline in the *Gammaproteobacteria* profile in weeks one to five. Identifiable *Alteromonadaceae* genera included *Alteromonas*, *Glaciecola* and *Paraglaciecola*. *Gammaproteobacteria* abundance is supplanted by *Deltaproteobacteria* groups; *Desulfobulbaceae*, *Desulfuromonadaceae* and *Desulfovibrionaceae* and *Bacteroidetes* groups; and *Prolixibacteraceae* (*Draconibacterium* and *Roseimarinus*), *Flavobacteriaceae* (*Lutibacter*) *and Marinilabiliaceae* (*Labilibacter*). Families within *Firmicutes* and *Verrucomicrobia* were active CAZyme secretors despite low apparent abundances, particularly *Peptococcaceae*, *Planococcaceae* and *Paenibacillaceae and Rubritaleaceae*.

## Discussion

We examined the process of surface level lignocellulose decomposition within a natural salt marsh environment demonstrating a framework wherein lignocellulose decomposition can be monitored in situ. Our data suggest a large proportion of the total native microbiome is lignocellulose responsive and capable of rapid colonisation and restructuring to take advantage of this annual influx of carbon. Our metaproteomic studies revealed an enrichment for activities that target linkages between lignin and polysaccharides as well as glycanohydrolases and a marked sparseness of oxidative enzymes that attack lignin.

It is notable that although the total biomass in our mesh bags was reduced by about 70% over a 16-week period, our results conform to a first order decay model alluded to in previous experiments [28, 67–69]. These previous studies suggest the majority of particulate decomposition occurs within the first year of entry into the system and proceeds through three phases: the leaching of soluble compounds, decomposition and a final refractory phase characterised by diminished rates of decomposition [28, 67–69]. Valiela et al. [67] suggest the refractory period is confined to decomposition rates below 0.4% day^−1^. In our study, the decomposition rates for the refractory period during weeks eight and 16 were observed to be 0.22 ± 0.087% day^−1^ and 0.176 ± 0.03% day^−1^ respectively, suggesting our experiment ran into the refractory period.

We assessed surface level, aerobic lignocellulose decomposition as it has been shown to be significantly more efficient than sub-surface decay [30]. Valiela et al. [30] explored the relative composition of *Spartina alterniflora* for 24 months beginning in winter. While the study findings are not directly comparable to our own due to differing location, start date and species of biomass, which significantly affects decomposition [69], there is an undeniable synchrony between the profiles of lignocellulose degradation in both studies. Both studies demonstrated an initial increase in cellulose and hemicellulose, sequentially followed by lignin degradation, then hemicellulose degradation. The hemicellulose degradation then coincides with cellulose degradation while lignin increases.

The relative enrichment in lignin, accepted as the most recalcitrant component of lignocellulose [18], suggests this biopolymer is not actively targeted for metabolism by the microbial community. Salt marsh sediments are known to be significantly enriched in lignin-derived high molecular weight polyphenols, with these increasing in concentration with depth [10, 11, 30, 70]. Conversely, the more biologically available polysaccharides reduce with depth as they are known to be preferentially targeted [71–73]. As lignin interpenetrates the core polysaccharides in the lignocellulosic matrix, it must be removed before the internal polysaccharides are accessible for digestion. Oxidative enzymes are the predominant mechanism exhibited in terrestrial systems to modify and degrade lignin, yet in our study, only AA2 family members were present at low abundances. These enzymes attack lignin moieties to modify the structure and it is unlikely they are responsible for cleaving high molecular weight phenolics that are observed in salt marsh sediments. These findings suggest that native salt marsh organisms have enzymes responsible for lignin modification that are not yet known or that they adopt other mechanisms able to facilitate access to the valuable sugars present in lignocellulose.

Instead, we note that the salt marsh meta-exo-proteome has a high representation of carbohydrate esterases (CE), particularly from family 1 (CE1). CE1 family enzymes function non-oxidatively to remove cinnamoyl and acetyl esters from xylans, disrupting the lignin-carbohydrate complex interface between hemicellulose and lignin, and hemicellulose and cellulose respectively [18, 74]. Lignin-carbohydrate complex linkages are thought to consist mainly of aryl ester (from ferulic acid to arabinose in grasses like *Spartina anglica*) and aryl ether bonds, hydrolysis of which decouples the lignin, exposing the surface of the remaining polysaccharides [75]. The CE1 family includes a range of esterases, especially those which hydrolyse ester links between arabinoxylans and ferulic and coumaric acid residues. Ferulic acid residues in arabinoxylans are particularly important in providing linkages between arabinoxylan chains and between arabinoxylans and lignin, thereby contributing significantly to lignocellulose recalcitrance [18, 76, 77]. CE1 also contains xylan acetylesterases that remove acetyl groups from arabinoxylan, having major impacts on their three dimensional conformation and ability to bind cellulose [78]. Previous compositional analysis of decomposed lignocellulose in salt marshes have revealed trans-ferulic acid was responsible for 57–82% of the total lignin loss which agrees with the mechanism identified in our study [79]. This indicates that the linkages holding lignin to the polysaccharides of lignocellulose may be major targets to allow GHs access to their substrates. We contend that this mechanism is favourable within salt marshes in contrast to terrestrial systems due to the liquid medium facilitating desorption of dissociated lignin macromolecules into the surrounding waters, circumventing the requirement for total deconstruction. This mechanism could explain the enrichment of persistent lignin-rich particles known to accumulate in salt marsh sediments through the cleavage of high molecular weight phenolics. These phenolics are then likely subject to oxidative modification by the low abundance AA2 family enzymes causing them to slowly become biologically available.

Previous studies suggest lignocellulose degradation within sediments is driven by bacteria, which is supported by our data [32, 34], yet fungi are known to populate salt marsh sediments but their function, community ecology and interactions remain elusive [80]. We did identify a handful of fungal families with potential historical connections to lignocellulose, predominantly *Pleosporaceae*, *Hypocreaceae*, *Nectriaceae*, *Sordariaceae* and *Saccharomycetales* [81]. Nutrient acquisition strategies of the identified fungi revealed the dominant trophic mode to be saprotroph (acquire nutrients from dead organic matter) and to a lesser extent pathotroph (acquire nutrients by attacking cells) and combinations thereof. This suggests most fungi were acquiring nutrients from alternative dead organic matter or were utilising a pathotrophic acquisition strategy where lignocellulose is not a primary target. A notable observation was that wood saprotrophs and leaf saptrotrophs, which would be expected to thrive on the dead *Spartina* biomass which included stems, leaves and sheaths, were present in the sediment but were not abundant on the lignocellulosic material. As fungi are orders of magnitude less abundant than bacteria in this system [82, 83] and we did not detect lignocellulolytic enzymes from these groups within the meta-exo-proteome despite fungal enzyme sequences being well represented in archive databases, our data would suggest their influence on lignocellulose decomposition for material within salt marsh sediments is negligible and they likely target alternative sources of organic matter that are present or cohabit within the lignocellulosic aggregate.

Bacterial families that have been implicated with salt marsh lignocellulose degradation based on isotope probe experiments include *Desulfobacteraceae*, *Spirochaetaceae*, *Kangiellaceae* [32] and selective enrichments include *Flavobacteriaceae*, *Cyclobacteriaceae*, *Pseudomonadaceae* and *Halomonadaceae* [34]. We did not observe the groups reported by Darjany et al. [32] to be active lignocellulose degraders, since the majority of lignocellulose deconstruction occurs within the extracellular matrix the breakdown products are available to all microbes within proximity, therefore the ^13^C approach employed by Darjany et al. [32] possibly identified benefactors of breakdown products rather than organisms actively degrading lignocellulose. We did identify all major groups reported by Cortes-Tolalpa et al. [34] in our in situ study which confirm these groups to be active secretors of lignocellulolytic enzymes. We also identified an additional 38 families that were not previously known to actively secrete lignocellulose-active enzymes. The 42 CAZyme-producing families reported here underpin long-term carbon sequestration using a mechanism that appears to favour the degradation of complex polysaccharides by selectively avoiding lignin degradation. This process not only expands the pool of stored carbon but also reduces complex carbohydrates to biologically available molecules within the extracellular space for the wider microbial community.

The CAZyme-producing *Gammaproteobacteria* described here appeared to be early colonisers of lignocellulose that undergo taxonomic restructuring to favour heterotrophic lineages. *Gammaproteobacteria* are displaced by CAZyme-producing groups belonging to *Bacteroidetes* and *Deltaproteobacteria* clades. The results suggest the *Gammaproteobacteria* families are the ecologically dominant surface level lignocellulose degraders. The divergent families identified within *Bacteroidetes* and *Deltaproteobacteria* suggest they are highly active at surface levels, but likely dominate carbon cycling in the oxygen-depleted cores of biomass aggregates and in shallow to deep sub-surface sediments as they have been identified in abundance within deeper sediments. However, their ecological functions were previously unknown [84].

Well studied examples of marine lignocellulolytic *Gammaproteobacteria* include *Saccharophagus degradans* and the closely related *Teredinibacter turnerae*, belonging to families *Alteromonadaceae* and *Cellvibrionaceae*, respectively. Both families were abundant within the lignocellulose responsive microbiome and identified to be highly productive of CAZymes and interestingly, neither family was well represented within the day 0 sediment outgroup suggesting they function as saprotrophs within the salt marsh. *S*. *degradans* is a well-characterised free-living heterotroph that appears fully capable of deconstructing complex plant cell wall polysaccharides and many other biopolymers [85, 86]. The use of these bacteria as a source of enzyme cocktails for lignocellulose saccharification has been explored due to the broad complement of CAZymes [87] and full cellulolytic system [88]. Dominant CAZymes within *S*. *degradans* culture supernatant include GH3, GH5, GH6, GH9, GH10 and GH16 many of which are multi-domain with a prevalence of CBM2 and CBM10 containing proteins as well as CBM6, CBM13 and CBM32 [89], all of which collectively correspond to highly abundant *Gammaproteobacteria*-associated CAZyme families identified within our exo-meta-proteomics.

*T*. *turnerae* is a facultative intracellular endosymbiont found in wood-boring bivalves, it is cellulolytic with demonstrated cellulose degrading capability and more recently it has been found to harbour a complex array of xylan degrading enzymes and lytic polysaccharide monooxygenases [90, 91], yet it possesses a relatively small repertoire of CAZYmes that only target woody plant biomass within its genome compared to *S*. *degradans* [92]. These are predominantly GH5, GH6 and GH11 that also include multi-domain proteins often associated with CBM5 and CBM10 domains that were well represented in our corresponding *Gammaproteobacteria* exo-meta-proteome [92–94]. The prevalence of multi-domain proteins containing CBMs, particularly within the cellulases, observed both within the well-characterised marine isolates and observed to be highly abundant within this study have been suggested to function as a tether as opposed to a catalytic enhancement [89]. Less than 40% of terrestrially derived cellulases contain CBMs that are conventionally thought to increase the effective concentration of enzyme at the substrate surface and therefore rates of activity [95]. In salt marshes, adsorption of CBM containing enzymes would act to tether and localise the enzyme to the substrate, improving substrate beneficiation to the secretor by preventing them from being washed away in these intertidal regions. It is possible that the predominance of CAZymes possessing CBMs has facilitated the *Gammaproteobacteria* to flourish within the early stage of surface level decomposition within the salt marsh as observed in both our exo-meta-proteome and independent 16S rRNA amplicon profile.

Carbohydrate-active enzymes are an incredibly broad designation of enzymes that includes both the intracellular and extracellular biosynthesis and breakdown of complex and simple polysaccharides. Accurately determining extracellular localisations of proteins from transcripts, particularly in an underexplored environment such as a salt marsh is challenging. Therefore, we applied exo-meta-proteomics to accurately determine proteins existing within the extracellular matrix. We successfully identified 81 CAZyme families within the exo-meta-proteome of the 103 identified within the metatranscriptome libraries suggesting sufficient depth of coverage of the extracellular encompassing families. However, due to the salt marsh ecosystem being intertidal, it is possible our analysis has not detected transiently localised enzymes. This may explain the small subset of predominantly pectin-targeting CAZyme families that were present within the transcriptome but not detected within the exo-meta-proteome as pectin is widely considered the most soluble component of the plant secondary cell wall.

Our approach targeted the rapid surface level deconstruction phase of lignocellulose. While the salt marsh microbiome varies marginally with elevation at the sediment surface [96], it is significantly variable with depth [84]. These changes are a function of oxygen depletion, leaching rates, sorption characteristics and alternative respiratory terminal electron acceptor availability. Considered together with the relative enrichment in lignin-derived polyphenolics [10, 11, 97, 98], this suggests the lignocellulose-active community could be stratified with depth. These communities may employ alternate mechanisms than identified here that target the most recalcitrant, lignin-enriched material that has once passed through the initial surface level decomposition phase we describe. Our results only capture the initial rapid surface level decomposition phase; these findings cannot be extrapolated throughout the salt marsh sedimentary column where the majority of the carbon stock persists. Further exploration of the microbial communities at depth is required to elucidate the functional taxa and the mechanisms they employ to degrade the lignin-enriched carbohydrate complexes that progressively accumulate and contribute to the extensive pool of sequestered carbon as substrate composition is known to modulate the mechanisms employed [99].

## Conclusions

Our study captured lignocellulolytic organisms as they functioned in situ at their environmental interface within surface sediments in a salt marsh. We identified 42 families that actively secrete enzymes that act to deconstruct lignocellulosic polymers, 38 of these families had no previously proven ecological function. Our data suggest that bacteria primarily orchestrate this process within sediments with no detectable contribution from fungi despite being present. Our proteomic analysis of the meta-exo-proteome highlighted *Gammaproteobacteria* as early lignocellulolytic colonisers that are temporally displaced by *Bacteroidetes and Deltaproteobacteria* groups and these taxa concurrently produce a core suite of diverse enzymes that act upon lignocellulose. This also revealed a potential mechanism of deconstruction, driven by carbohydrate esterase family 1 enzymes, which are capable of dissociating lignin macromolecules from the core polysaccharides within the lignocellulosic complex. This degradative strategy potentially explains the accretion of lignin-derived polyphenolics within salt marsh sediments. As our study assessed early stage surface level degradation, further research is required to elucidate mechanisms that drive organic carbon storage and turnover in deeper sediments.

## Supplementary Information

**Additional file 1: Figure S1.** Experiment location. **Figure S2.** Coverage estimates for each 16S rRNA amplicon library for all biological replicates across each time point. **Figure S3.** CAZyme families identified within the metatranscriptomic databases presented as transcripts per million. **Figure S4.** CAZyme producing genera and their respective CAZyme contributions. **Figure S5.** Productivity index for CAZyme producing taxa at family level resolution. **Figure S6. **Phylogenetic distribution of CAZyme classes at class resolution for week one. **Figure S7.** Phylogenetic distribution of CAZyme classes at class resolution for week three. **Figure S8.** Phylogenetic distribution of CAZyme classes at class resolution for week five. **Figure S9.** Phylogenetic distribution of CAZyme classes at class resolution for week ten. **Figure S10.** Functional classification of proteins within the metasecretome. **Figure S11.** Fungal profiles and OTU richness elucidated from internal transcribed spacer region 2 amplicon sequencing across the 16-week time course. **Figure S12.** Nutrient acquisition strategy of fungi profile across the 16-week time course. **Figure S13.** Bacteria profiles elucidated from 16S rRNA sequence homology. **Figure S14.** Bacteria profiles elucidated from 16S rRNA sequence homology. **Table S1.** Transect position of the five biological replicates in Welwick salt marsh. **Table S2.** Sequence reads throughout RNA sequence processing and assembly. **Table S3. **Commands for the replication of the 16S rRNA amplicon database processing pipeline.

**Additional file 2.** Molar percentages. dbCAN annotations. Taxonomic homology.

## Data Availability

Metaproteomic and metatranscriptomic databases generated during this research are available at MassIVE (https://massive.ucsd.edu/) MSV000083872 and ProteomeXchange (http://www.proteomexchange.org/) PXD014068. The raw 16S rRNA gene sequencing data generated and analysed in this study is available from the European Nucleotide Archive (https://www.ebi.ac.uk/ena) under accessions PRJEB32810 and ERP115532. A curated dataset is available in the supplemental dataset found in Additional file 2. In-house scripts, datasets and dependencies for reproducing this analysis are available at https://github.com/leadbot/Salt-marsh-metasecretome-analysis.

## Abbreviations

AA: Auxiliary activity enzyme
CAZyme: Carbohydrate-active enzymes
CBM: Carbohydrate-binding module
CE: Carbohydrate esterase
GH: Glycoside hydrolase
ITS2: Internal transcribed spacer region 2
LC-MS/MS: Liquid chromatography with tandem mass spectrometry
ORF: Open reading frame
OTUs: Operational taxonomic units
PBS: Phosphate-buffered saline
PCR: Polymerase chain reaction
PL: Polysaccharide lyase
TPM: Transcripts per million
