# Supplementary material for: Mechanistic strategies of microbial communities regulating lignocellulose deconstruction in a UK salt marsh
Source: Microbiome. 2021 Feb 17;9:48. doi: 10.1186/s40168-020-00964-0 (PMC7890819; doi:10.1186/s40168-020-00964-0)
Supplement: Supplementary file 2 — Additional file 1: Figure S1. Experiment location. Figure S2. Coverage estimates for each 16S rRNA amplicon library for all biological replicates across each time point. Figure S3. CAZyme families identified within the metatranscriptomic databases presented as transcripts per million. Figure S4. CAZyme producing genera and their respective CAZyme contributions. Figure S5. Productivity index for CAZyme producing taxa at family level resolution. Figure S6. Phylogenetic distribution of CAZyme classes at class resolution for week one. Figure S7. Phylogenetic distribution of CAZyme classes at class resolution for week three. Figure S8. Phylogenetic distribution of CAZyme classes at class resolution for week five. Figure S9. Phylogenetic distribution of CAZyme classes at class resolution for week ten. Figure S10. Functional classification of proteins within the metasecretome. Figure S11. Fungal profiles and OTU richness elucidated from internal transcribed spacer region 2 amplicon sequencing across the 16-week time course. Figure S12. Nutrient acquisition strategy of fungi profile across the 16-week time course. Figure S13. Bacteria profiles elucidated from 16S rRNA sequence homology. Figure S14. Bacteria profiles elucidated from 16S rRNA sequence homology. Table S1. Transect position of the five biological replicates in Welwick salt marsh. Table S2. Sequence reads throughout RNA sequence processing and assembly. Table S3. Commands for the replication of the 16S rRNA amplicon database processing pipeline. [file 40168_2020_964_MOESM2_ESM.docx]

Supplementary Information for:

Mechanistic strategies of microbial communities regulating lignocellulose deconstruction in a UK salt marsh

**Authors**

Daniel R. Leadbeater^1§^, Nicola C. Oates^1^, Joseph P. Bennett^1^, Yi Li^1^, Adam A. Dowle^2^, Joe D. Taylor^4^, Juliana Sanchez Alponti^1^, Alexander T. Setchfield^1^, Anna M. Alessi^1^, Thorunn Helgason^3^, Simon J. McQueen-Mason^1§^, Neil C. Bruce^1§^

^1^ Centre for Novel Agricultural Products, Department of Biology, University of York, York, YO10 5DD, UK.

^2^ Bioscience Technology Facility, Department of Biology, University of York, York, YO10 5DD, UK.

^3^ Department of Biology, University of York, York, YO10 5DD, UK.

^4^ School of Chemistry and Biosciences, University of Bradford, Bradford, West Yorkshire, BD7 1DP, UK.

^§^ Corresponding authors: N.C.B. (email: neil.bruce@york.ac.uk), S.J.M.-M. (email: simon.mcqueenmason@york.ac.uk) or D.R.L. (email: daniel.leadbeater@york.ac.uk).


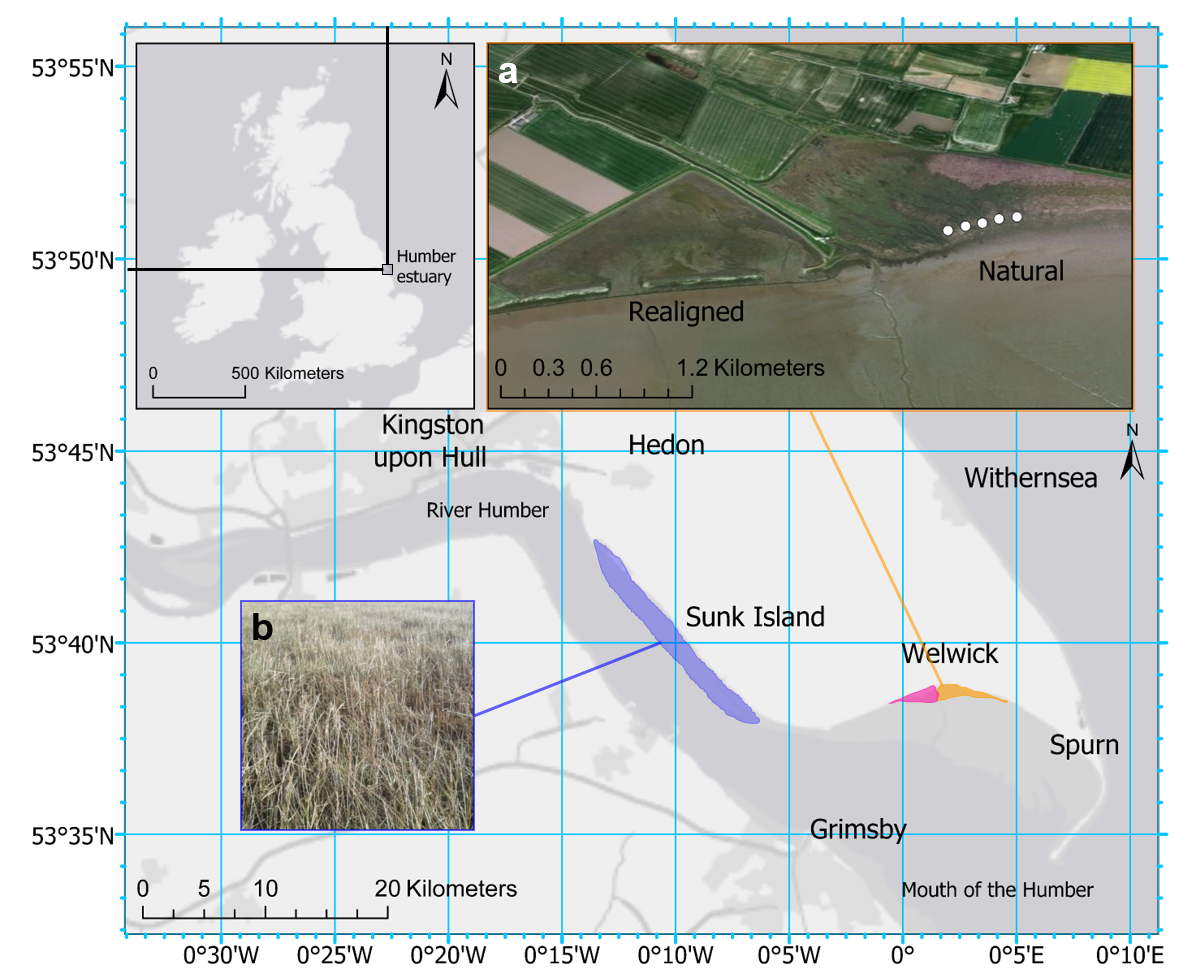


**Figure S1.** Experiment location. The Humber estuary (Hull, UK). The North bank of the Humber estuary, Welwick salt marsh (inlet a) is highlighted in orange and the cage locations along the transect in white within the inlet. Cherry Cobb Sands is highlighted in blue with the location of the collected Spartina anglica (inlet b).


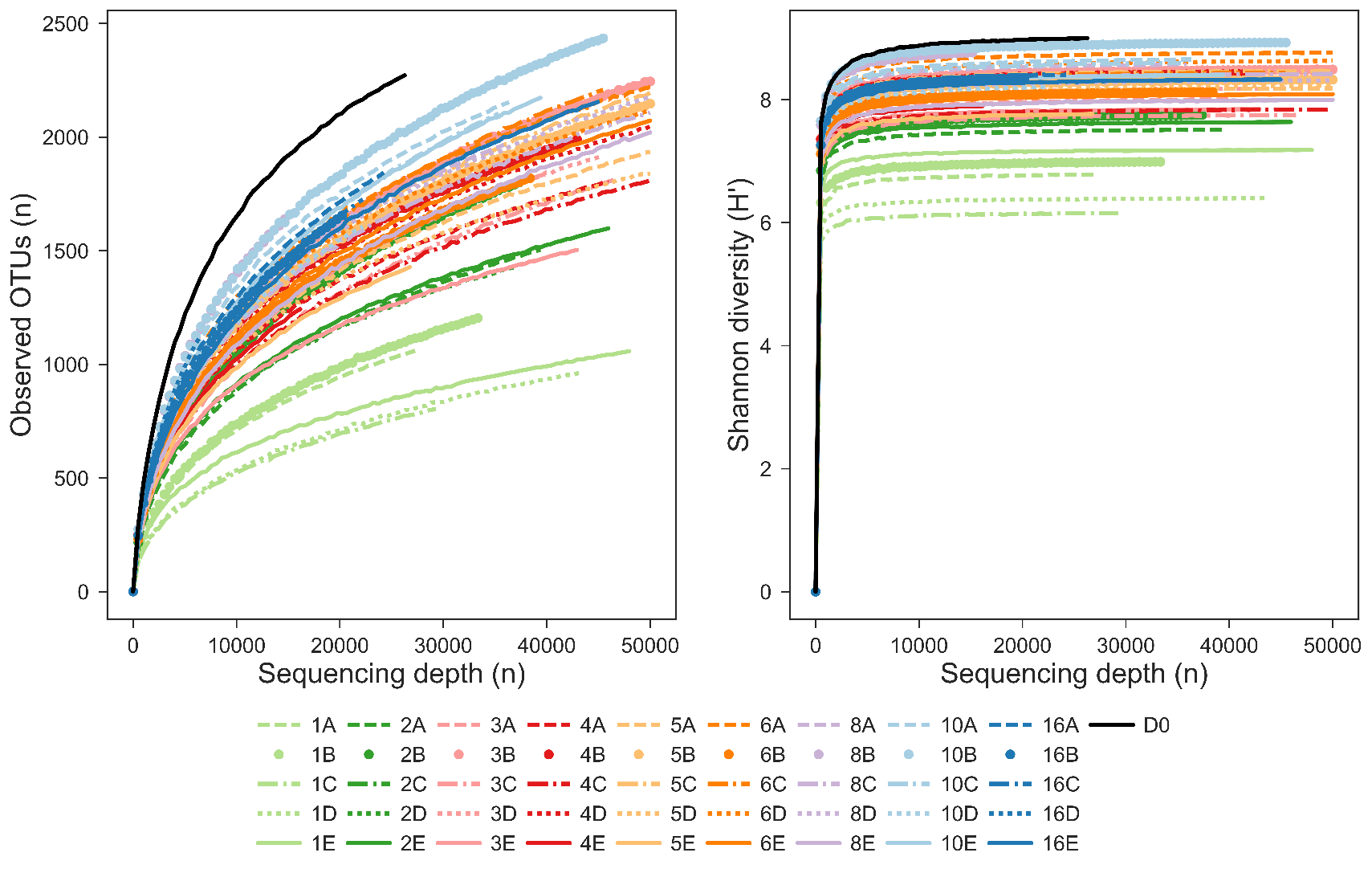


**Figure S2.** Coverage estimates for each 16S rRNA amplicon library for all biological replicates across each time point. **a;** Rarefaction curves identified by number of observed operational taxonomic units (OTUs) and sequencing depth. **b;** Shannon diversity index collector curves as a function of sequencing depth.


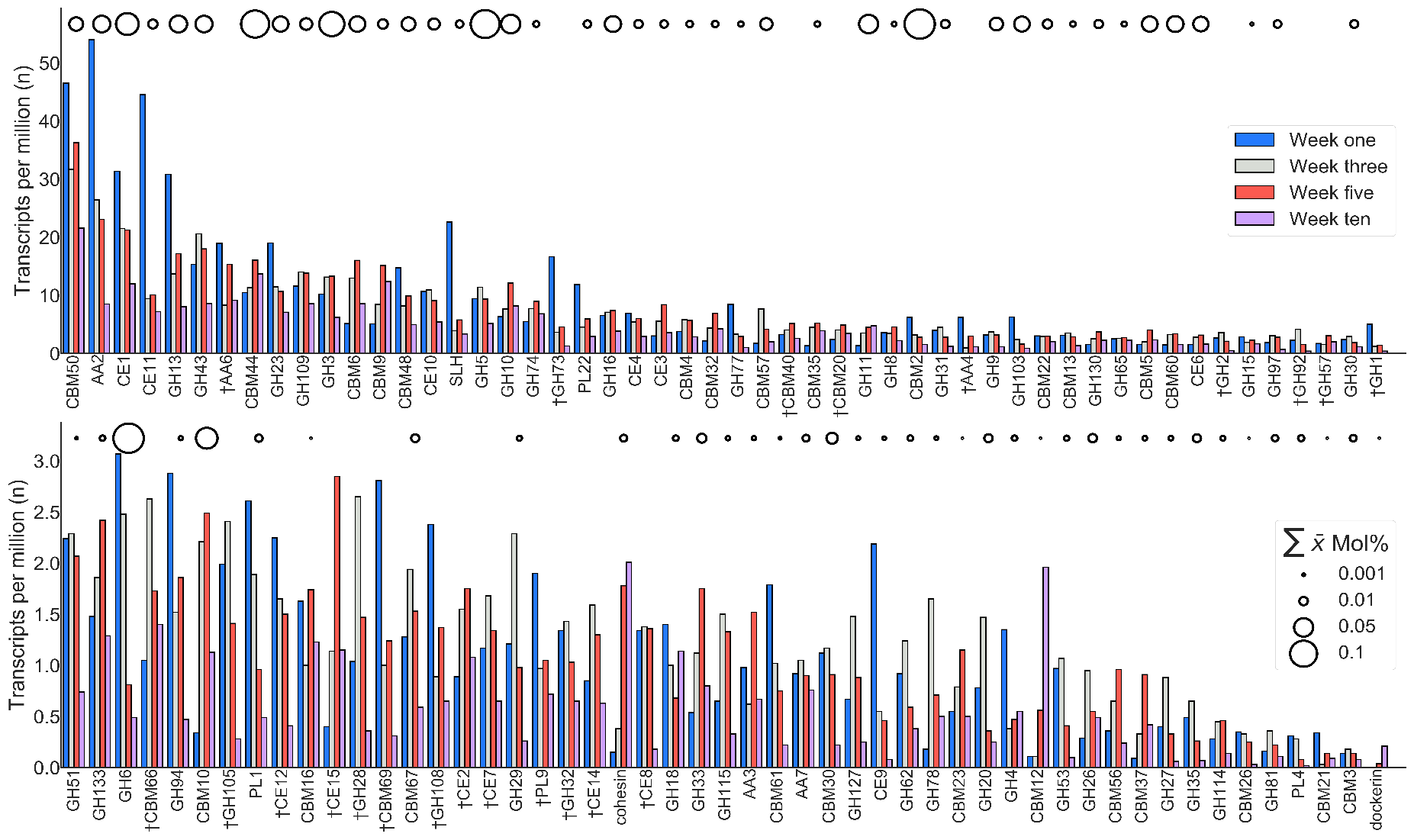


**Figure S3.** CAZyme families identified within the metatranscriptomic databases presented as transcripts per million (TPM). Glycosyl transferase families have been filtered and only annotations with expect values ≤ 1e^-5^ and TPM values ≥ 1 have been included for analysis. **†** delineates the CAZyme family was not identified within the meta-exo-proteome. Marker size is proportional to the mean sum of the molar percentage across the exo-meta-proteome for each week. *GH:* Glycoside hydrolase, *CE:* Carbohydrate esterase, *AA:* Auxiliary activity, *PL:* Polysaccharide lyase, *CBM:* Carbohydrate binding domain. Transcriptomic data includes both intracellular and extracellular representatives of the respective CAZyme families.


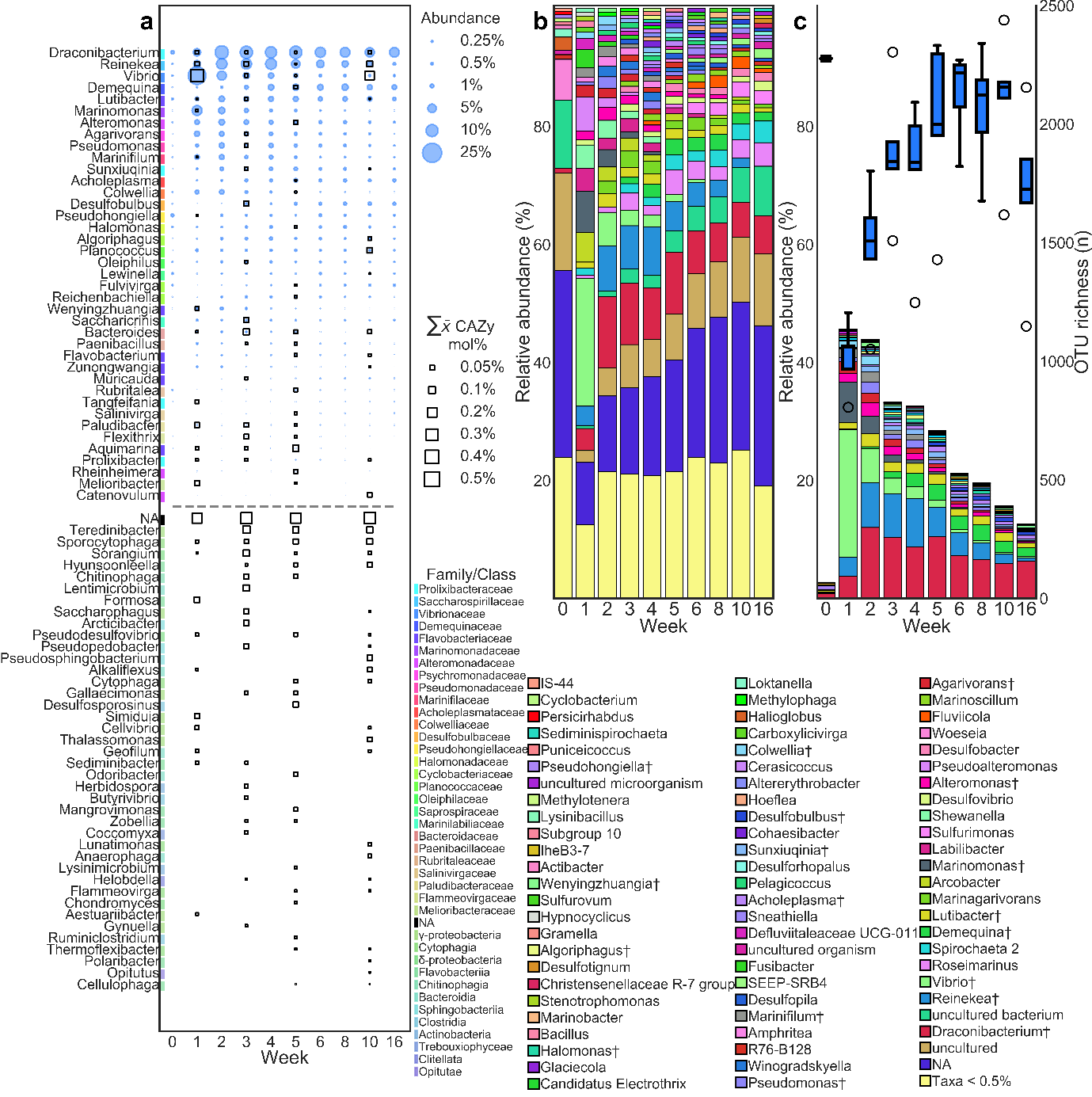


**Figure S4.** CAZyme producing genera and their respective CAZyme contributions. Microbiome and proteomic data is displayed as the mean of n=5 and n=3 respectively. **a;** Distribution of CAZyme producing lineages with respective CAZyme productivity (≤ 1e^-10^), taxa below the dashed line were not identified in the community profile, ordered by descending abundance by total temporal sum. **b;** Bacteria profiles elucidated from 16S rRNA sequence homology, each time point is the mean of five biological replicates. **c;** CAZyme productive bacteria profile, the non-CAZyme productive taxa have been filtered, boxes display OTU richness, no further filtering was undertaken for these data. *NA:* Not assigned, *†:* CAZyme producer.


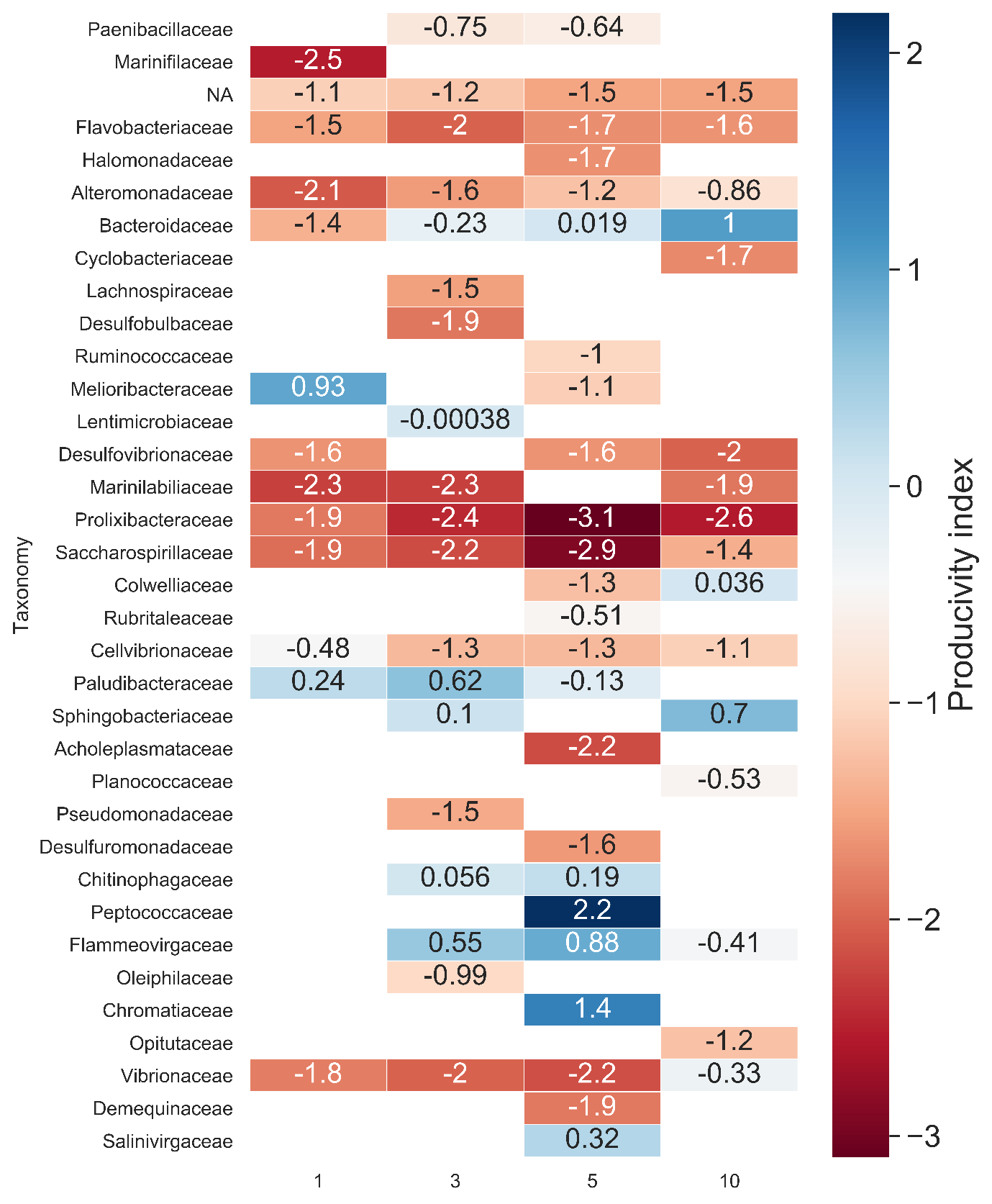


**Figure S5.** Productivity index for CAZyme producing taxa at family level resolution. A higher index indicates disproportionately more CAZyme produced per unit abundance. Values displayed as log­­­_10_(∑ x̄ mol%/Relative abundance).


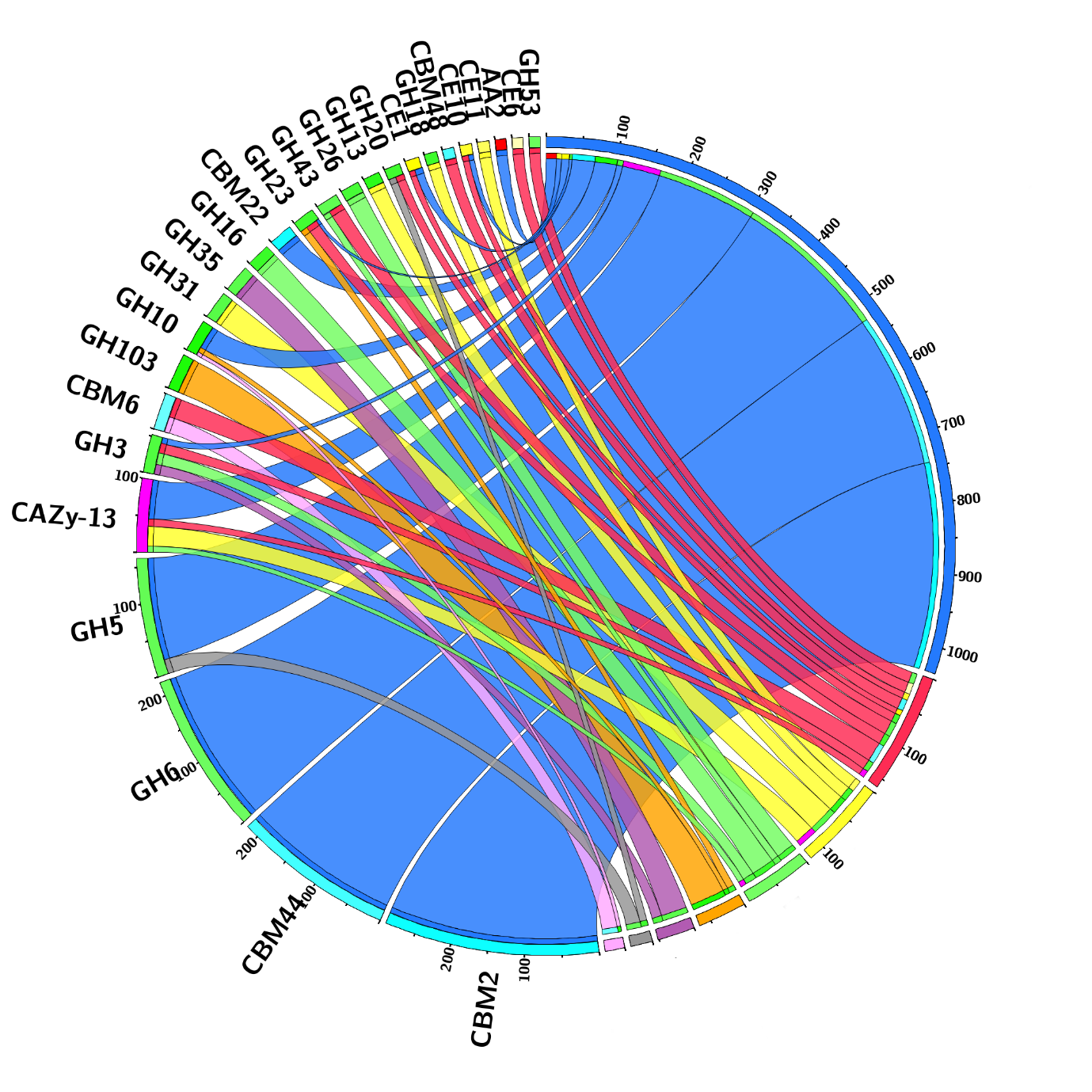


**
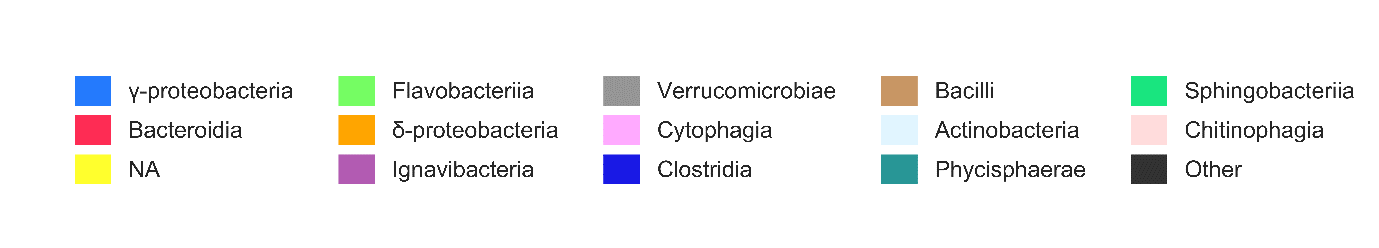
Figure S6.** Phylogenetic distribution of CAZyme classes at class resolution for week one. Absolute values of segments are the sum molar percentage 1e^3^ (1000 = 1%). Ribbon size is relative to the contribution to or of the taxa/ CAZyme and are only interspecifically comparable. Taxa producing < 1% of the total CAZyme and CAZyme classes < 0.85% of the total have been filtered, no further proteomic filtering criteria was applied to this data. Chord diagrams were generated using the Circos software package [1]. *NA:* Not assigned.

**
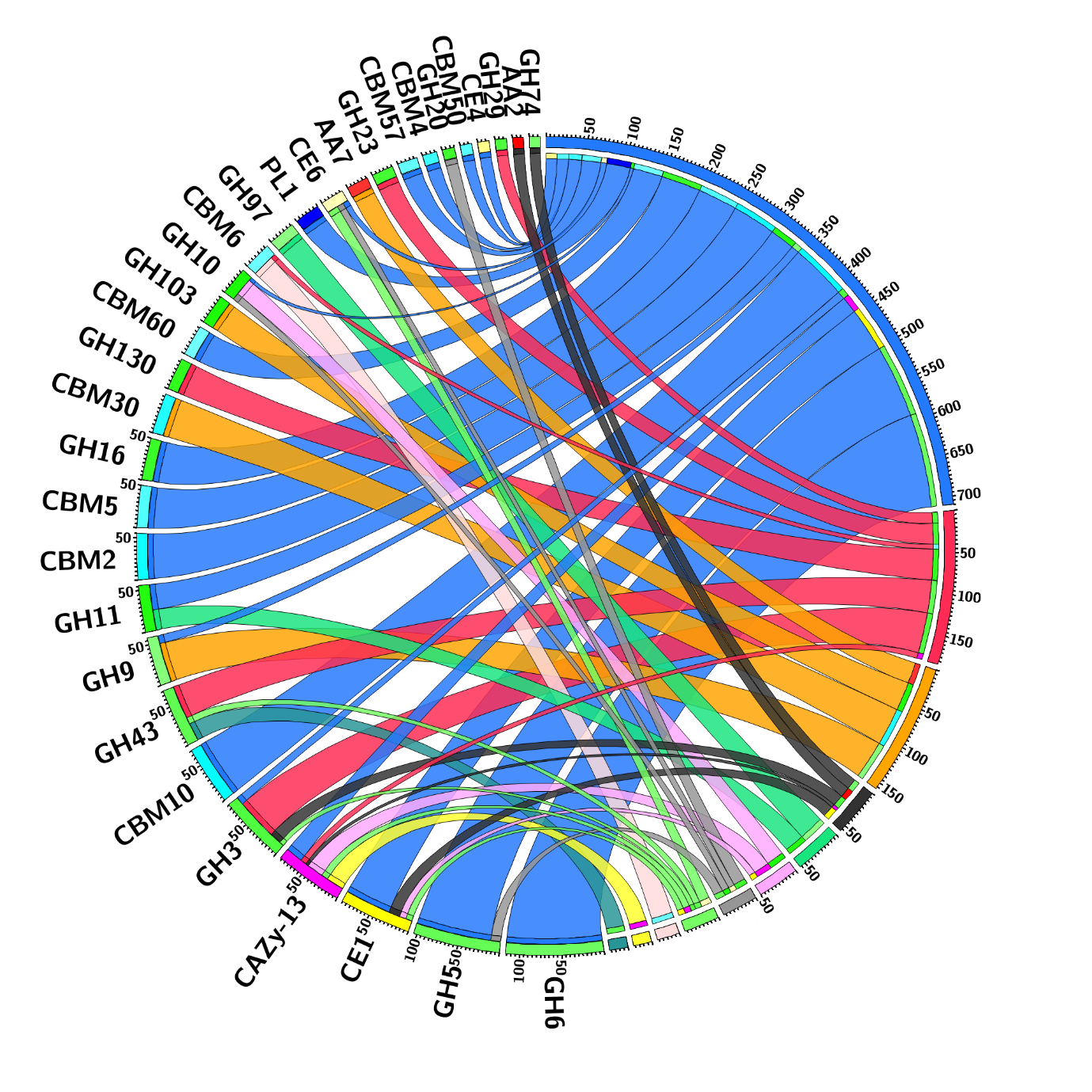
**

**
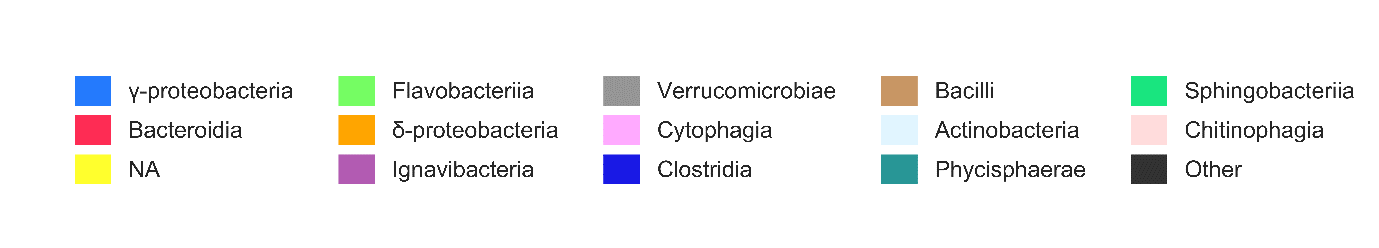
**

**Figure S7.** Phylogenetic distribution of CAZyme classes at class resolution for week three. Absolute values of segments are the sum molar percentage 1e^3^ (1000 = 1%). Ribbon size is relative to the contribution to or of the taxa/ CAZyme and are only interspecifically comparable. Taxa producing < 1% of the total CAZyme and CAZyme classes < 0.85% of the total have been filtered, no further proteomic filtering criteria was applied to this data. Chord diagrams were generated using the Circos software package [1]. *NA:* Not assigned.


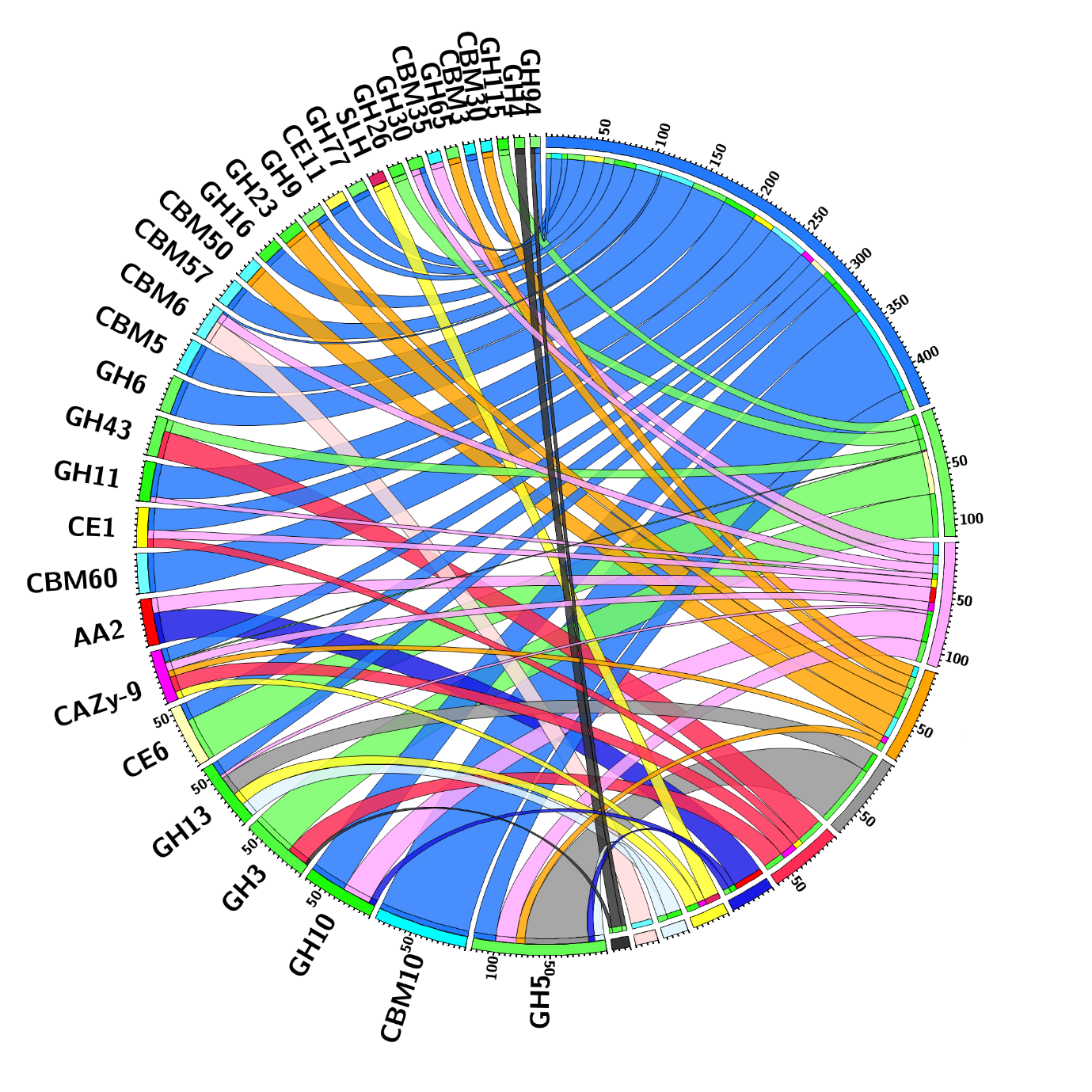


**
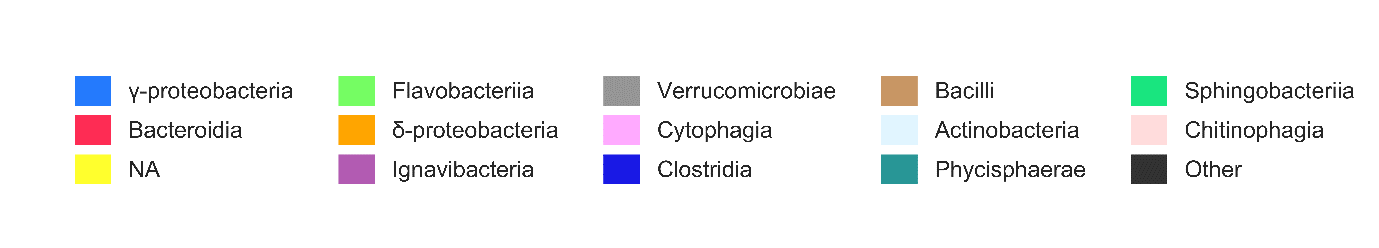
**

**Figure S8.** Phylogenetic distribution of CAZyme classes at class resolution for week five. Absolute values of segments are the sum molar percentage 1e^3^ (1000 = 1%). Ribbon size is relative to the contribution to or of the taxa/ CAZyme and are only interspecifically comparable. Taxa producing < 1% of the total CAZyme and CAZyme classes < 0.85% of the total have been filtered, no further proteomic filtering criteria was applied to this data. Chord diagrams were generated using the Circos software package [1]. *NA:* Not assigned.


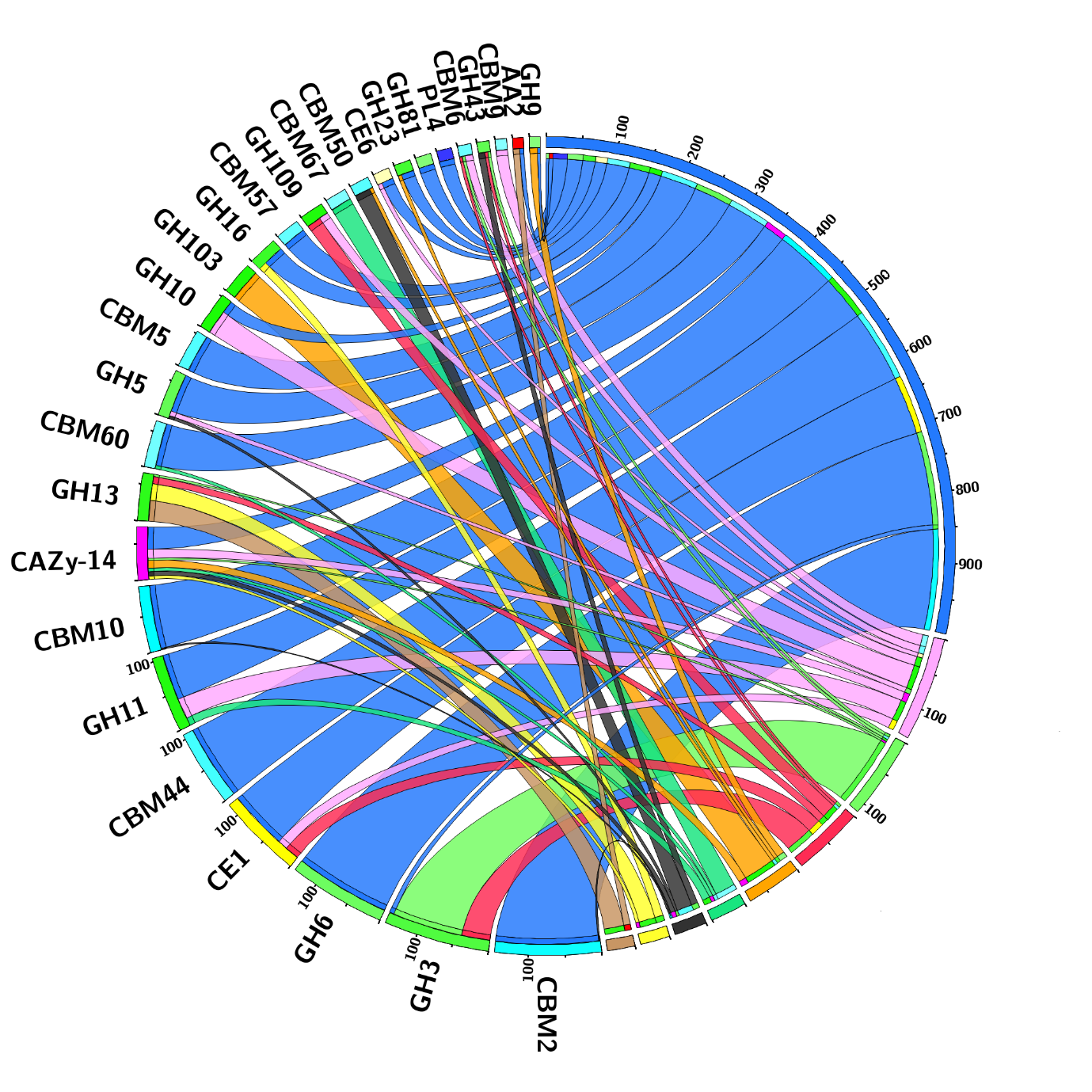


**
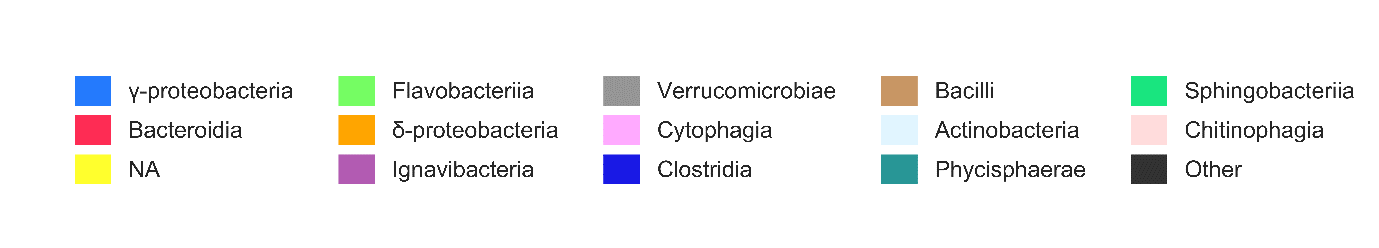
**

**Figure S9.** Phylogenetic distribution of CAZyme classes at class resolution for week ten. Absolute values of segments are the sum molar percentage 1e^3^ (1000 = 1%). Ribbon size is relative to the contribution to or of the taxa/ CAZyme and are only interspecifically comparable. Taxa producing < 1% of the total CAZyme and CAZyme classes < 0.85% of the total have been filtered, no further proteomic filtering criteria was applied to this data. Chord diagrams were generated using the Circos software package [1]. *NA:* Not assigned.


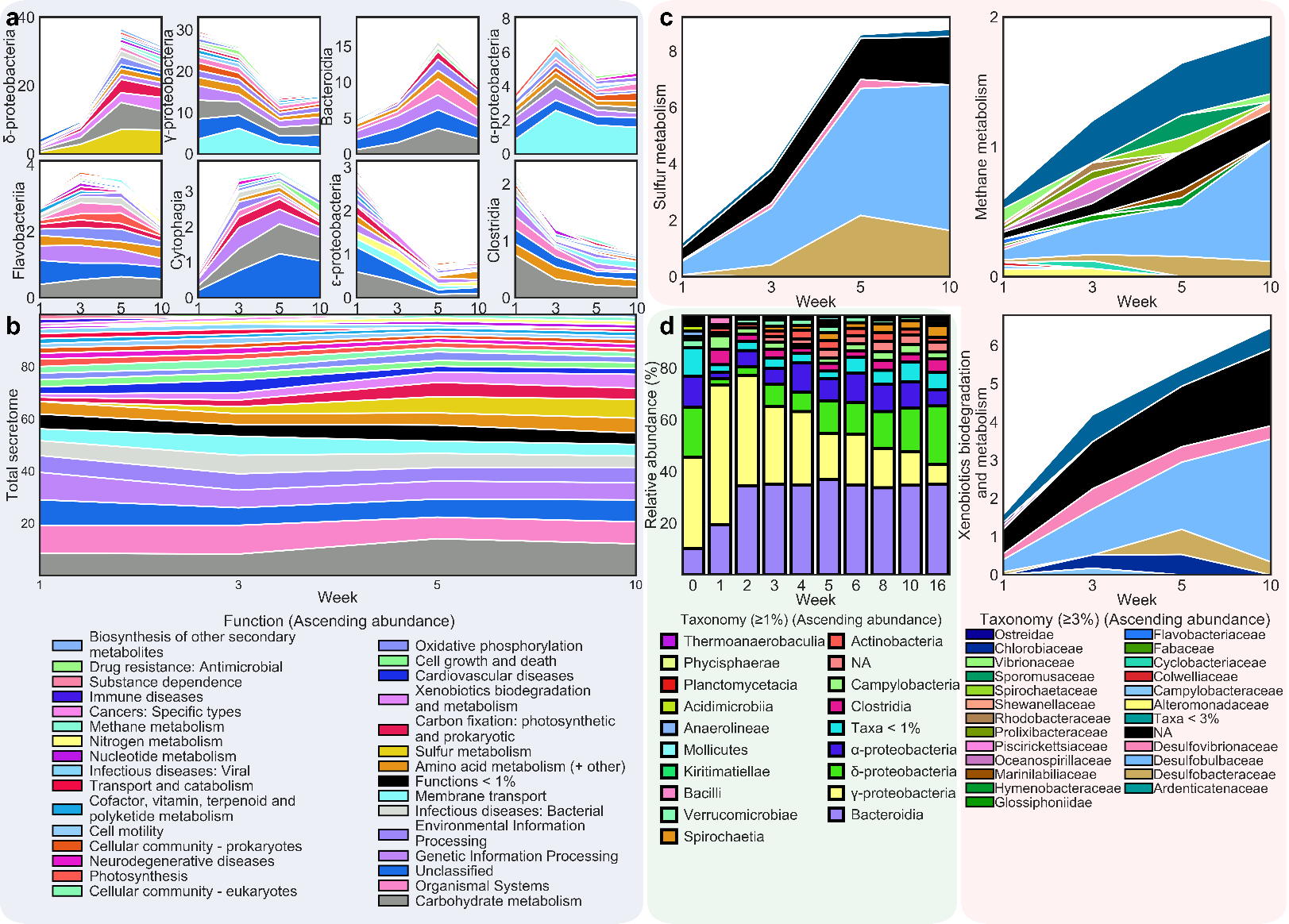


**Figure S10.** Functional classification of proteins within the metasecretome. Unclassified orthologs are not displayed. One to many mapping applied. **a;** Temporal distribution of functional KEGG orthologs [2] attributable to taxonomic classes (∑ x̄ mol%.) **b;** Functional assignment of the total metasecretome (∑ x̄ mol%). **c;** Taxonomic distribution of major functional pathways identified from the metasecretome (∑x̄ mol%). **d;** Bacteria profile. *NA:* Not assigned.


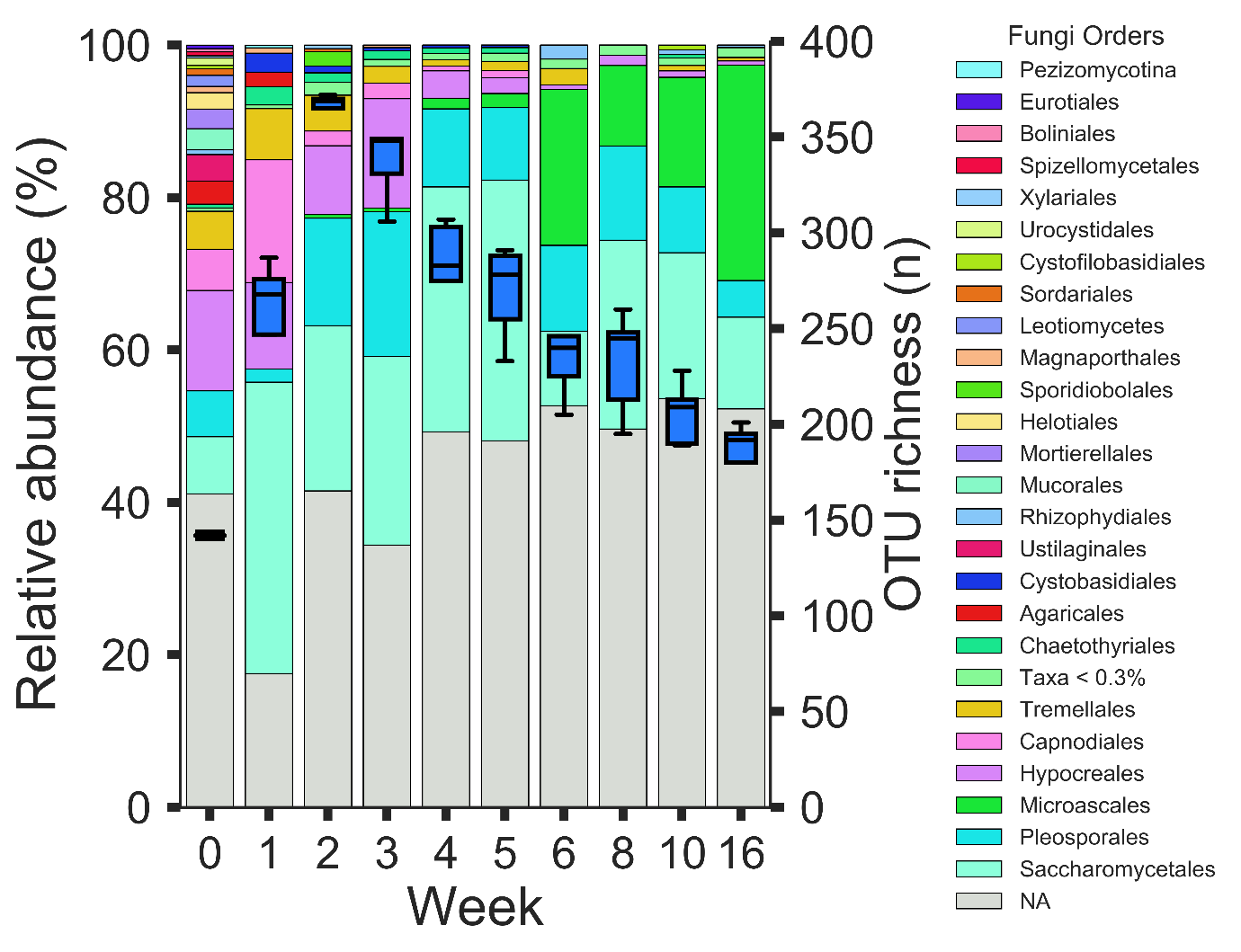


**Figure S11.** Fungal profiles and OTU richness elucidated from internal transcribed spacer region 2 amplicon sequencing across the 16-week time course. Data is displayed as the mean of n=5.


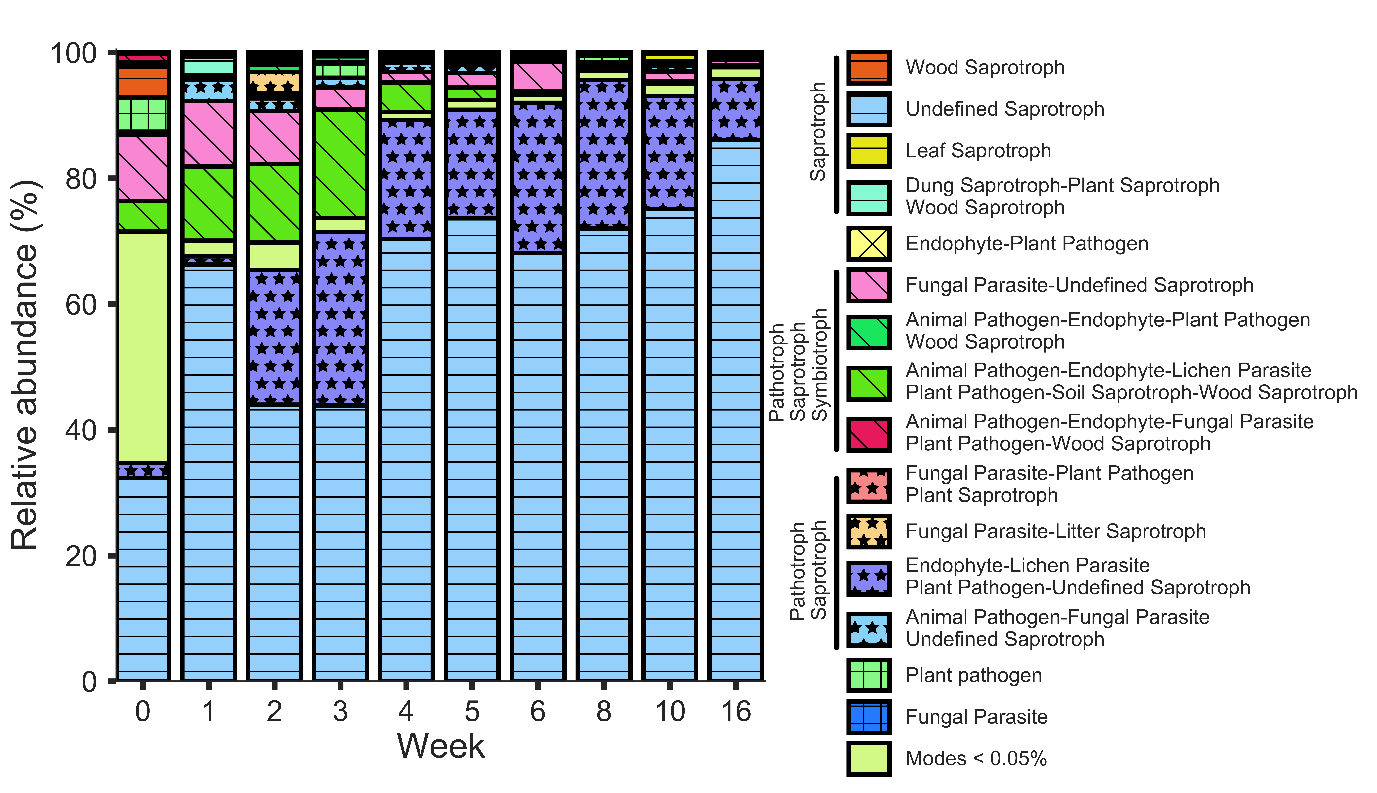


**Figure S12.** Nutrient acquisition strategy of fungi profile across the 16-week time course. Data is displayed as the mean of n=5. Guild and trophic mode are displayed.


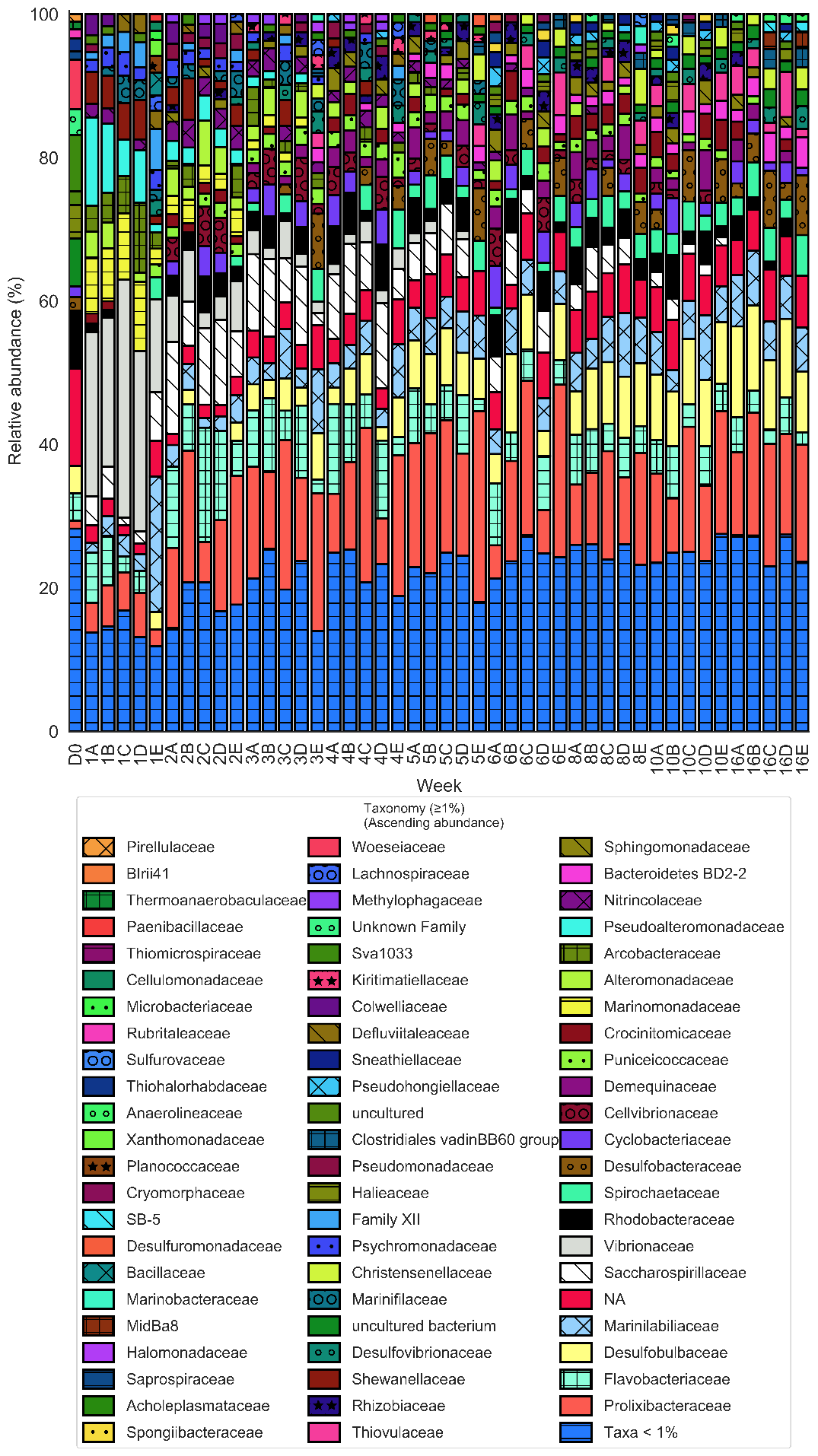


**Figure S13.** Bacteria profiles elucidated from 16S rRNA sequence homology, all biological replicates are displayed at family level.


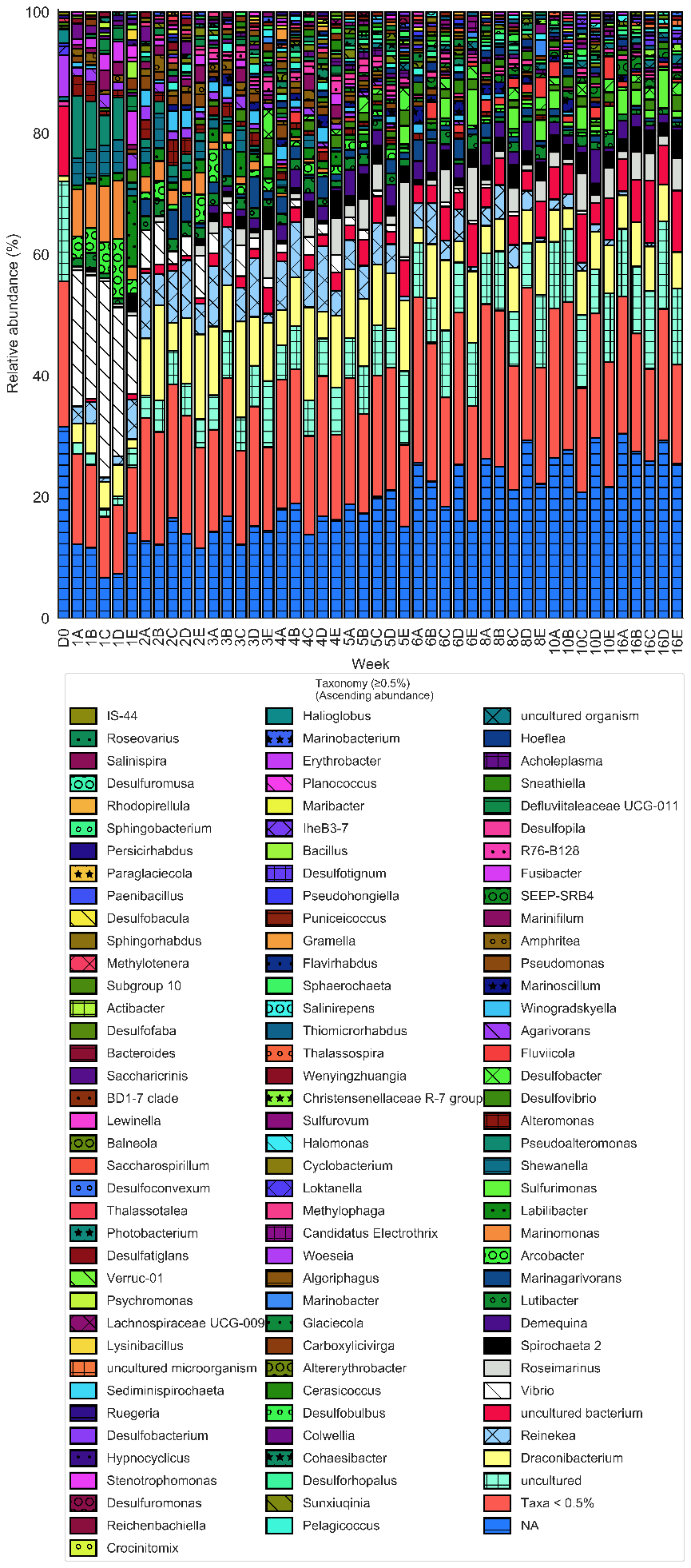


**Figure S14.** Bacteria profiles elucidated from 16S rRNA sequence homology, all biological replicates are displayed at genus level.

**Table S1.** Transect position of the five biological replicates in Welwick salt marsh. *OS GR*: ordnance survey grid reference.

| **Cage** | **OS GR** | **Coordinates** | **Latitude/Longitude** | **Elevation (m)** |
| --- | --- | --- | --- | --- |
| **A** | TA 33850 18626 | 53.647022, 0.023465794 | 53°38'49.28"N/ 0° 1'24.48"E | 2 |
| **B** | TA 33915 18656 | 53.647271,0.024455525 | 53°38'50.18"N/ 0° 1'28.04"E | 2.2 |
| **C** | TA 33977 18676 | 53.647439, 0.025407313 | 53°38'50.78"N/ 0° 1'31.47"E | 2.7 |
| **D** | TA 34038 18704 | 53.647678,0.026343800 | 53°38'51.64"N/ 0° 1'34.84" | 3 |
| **E** | TA 34105 18719 | 53.647792, 0.027360971 | 53°38'52.05"N/ 0° 1'38.50"E | 3 |

† Coordinate accuracy ±4m

**Table S2.** Sequence reads throughout RNA sequence processing and assembly.

|  | Week 1 | Week 3 | Week 5 | Week 10 | Total |
| --- | --- | --- | --- | --- | --- |
| Raw reads | 82 966 972 | 99 319 322 | 95 318 915 | 105 517 252 | 383 122 461 |
| Percentage reads rRNA | 10.71% | 1.58% | 2.74% | 2.04% | - |
| Paired end reads (-rRNA, -duplicates) | 68 307 441 | 91 948 286 | 85 685 871 | 93 709 355 | 339 650 953 |
| Paired end reads (-post QC) | 68 302 150 | 91 926 491 | 85 676 818 | 93 700 742 | 339 606 201 |
| Contigs (Trinity) | **4 100 865** | **9 720 697** | **7 501 904** | **8 615 202** | **29 938 668** |
| Genes (Trinity) | 3 869 004 | 9 132 509 | 7 071 997 | 8 153 028 | 28 226 538 |
| Median contig length (bp) | **187** | **195** | **185** | **201** | - |
| Average contig length (bp) | **268.28** | **247.46** | **255.67** | **249.49** | - |

**Table S3.** Commands for the replication of the 16S rRNA amplicon database processing pipeline. Emboldened characters are specific input or output files. Only steps utilising third party software is shown, custom steps are not shown. A1-A12 represent input or output files.

| Step | Function | Command |
| --- | --- | --- |
| 1 | Merge | vsearch --fastq_mergepairs **X**1_001.fastq.gz --reverse **X**2_001.fastq.gz --fastqout **X**_16Smerged |
| 2A | Trim adapter | cutadapt -o nexteraremoved/**X** -a CTGTCTCTTATACACATCTGACGCTGCCGACGA **X** |
| 2B | Trim 13N | cutadapt --cut 13 -o **X X** |
| 3 | Fastq split | convert_fastaqual_fastq.py -c fastq_to_fastaqual -f **X** |
| 4 | Demultiplex | split_libraries.py -f **X** --max_ambig 0 -r -k -B -H 10 -M 2 -e 2 -b 7 -o **X** -m **MAP** |
| 5 | Trim primer | cutadapt --cut 19 --cut -20 -o **X X** |
| 6 | Concatenate | cat *.fna > catfile.fna |
| 7 | Format header | Format headers to >barcode label=sample_id;sequence_number_integer - custom |
| 8 | Global trim | usearch_v9 -fastx_truncate **A1** -trunclen 250 -fastaout **A2** |
| 9 | Dereplicate | usearch_v7 --derep_fulllength **A2** --output **A3** --log=log --sizeout --minuniquesize 2 |
| 10 | Sort by size | Usearch_v7 -sortbysize **A3** -output **A4** -minsize2 |
| 11 | Cluster | usearch_v9 -cluster_otus **A4**_cat16S.fna -otus **A5** -minsize 2 |
| 12 | Relabel | fasta_number.py **A5** OTU_>**A6** |
| 13 | Map OTUs | Usearch_v7 -usearch_global **A1** -db **A6** -strand plus -id 0.95 -uc A7 |
| 14 | Assign taxonomy | Assign_taxonomy.py -i **A6** -o **A8** --similarity 0.9 -r ref_set.fna -t ref_set.txt |
| 15 | OTU table (.txt) | uc2otutab.py **A7** > **A9**.txt |
|  |  |  |
| 16* | .biom | Biom convert -I **A9** -o **A10** --table-type="OTU table" --to-hdf5 |
| 17* | .tsv | Biom convert -i **A10** -o **A11** --to-tsv |
| *Optional |  |  |

**References**

1.Krzywinski M, Schein J, Birol I, Connors J, Gascoyne R, Horsman D, Jones SJ, Marra MA: Circos: An information aesthetic for comparative genomics. Genome Research. 2009; 19**:**1639-1645.

2.Kanehisa M, Sato Y, Morishima K: BlastKOALA and GhostKOALA: KEGG Tools for Functional Characterization of Genome and Metagenome Sequences. Journal of Molecular Biology. 2016;428:726-731.
